# PDGFR**β** signaling restrains myocyte function to limit the regenerative capacity of skeletal muscle

**DOI:** 10.1172/JCI188272

**Published:** 2025-12-16

**Authors:** Siwen Xue, Abigail M. Benvie, Jamie E. Blum, Benjamin D. Cosgrove, Anna E. Thalacker-Mercer, Daniel C. Berry

**Affiliations:** 1 The Division of Nutritional Sciences, Cornell University, Ithaca, New York, USA.; 2 Department of Cell, Development, and Integrative Biology, University of Alabama at Birmingham, Birmingham, Alabama, USA.; 3 Meinig School of Biomedical Engineering, Cornell University, Ithaca, New York, USA.

**Keywords:** Development, Muscle biology, Adult stem cells, Signal transduction, Skeletal muscle

## Abstract

Muscle cell fusion is critical for the formation and maintenance of multinucleated myotubes during skeletal muscle development and regeneration. However, the molecular mechanisms directing cell-cell fusion are not fully understood. Here, we identified platelet-derived growth factor receptor β (PDGFRβ) signaling as a key modulator of myocyte function in adult muscle cells. Our findings demonstrated that genetic deletion of *Pdgfrb* enhanced muscle regeneration and increased myofiber size, whereas *Pdgfrb* activation impaired muscle repair. Inhibition of PDGFRβ activity promoted myonuclear accretion in both mouse and human myotubes, whereas PDGFRβ activation stalled myotube development by preventing cell spreading to limit fusion potential. Furthermore, PDGFRβ activity cooperated with TGF-β signaling to regulate myocyte size and fusion. Mechanistically, PDGFRβ signaling required STAT1 activation, and blocking STAT1 phosphorylation enhanced myofiber repair and size during regeneration. Collectively, PDGFRβ signaling acts as a regenerative checkpoint and represents a potential clinical target to improve skeletal muscle repair.

## Abstract 2

Blocking a specific protein, PDGFRβ, enhances muscle myofiber repair and regeneration, whereas activating it slows down recovery by limiting cell size and fusion.

## Introduction

Adult skeletal muscle contains numerous myofibers supporting posture, movement, and metabolism. The development and maintenance of these fibers rely on the fusion of mononucleated muscle stem cells (MuSCs) or satellite cells (1, 2). MuSCs reside in a quiescent state surrounding the myofibers, but upon stress or injury, they can become activated (3–7). Activated MuSCs can self-renew or become transamplifying myoblasts, which differentiate into fusion-competent myocytes. Myocytes can then fuse with other myocytes or with existing myofibers, effectively repairing the injured myofibers (1, 3–9).

Cell fusion is a multistep process that requires cell-cycle exit, migration, and cell-cell interactions, and it occurs across diverse tissues and organisms (10–12). Additionally, fusogenic cells undergo significant plasma membrane remodeling and actin cytoskeleton reorganization, creating fusion protrusions and synapses that allow cell merging (10–18). Although myocytes express generalized fusion machinery, they also express unique muscle-specific fusogens such as myomaker and myomerger (myomixer/minion) (19–24). Even though myomaker and myomerger are necessary for myocyte merging, increasing their expression does not always enhance myocyte fusion and regeneration (25). This highlights the requirement for multiple regulatory and remodeling pathways for efficient fusion. In agreement with this notion, TGF-β signaling prevents fusion by modulating WNT/β-catenin signaling pathways and several actin-cytoskeleton regulatory genes (26–28). Nevertheless, the full spectrum of signaling pathways that facilitate myocyte membrane and actin cytoskeleton remodeling to coordinate fusion remains largely unidentified.

Platelet-derived growth factor receptor β (PDGFRβ), a receptor tyrosine kinase, has been shown to regulate cell proliferation, migration, and differentiation in various tissues (29–32). Canonical PDGFRβ signaling within pericytes and smooth muscle cells controls vascular integrity and promotes blood vessel formation and expansion (29, 33). In skeletal muscle, pericytes are critical for skeletal muscle development by promoting myocyte differentiation and limiting MuSC quiescence (34, 35). Several lineage studies have shown that *Pdgfrb*-marked cells can serve as myogenic precursor cells that regenerate muscle tissue after injury (35–38). More specifically, in vitro, PDGF ligands can stimulate myoblast amplification, with inconsistent observations regarding myotube development (39–42). Recent work has extended these observations by showing that PDGFRβ is preferentially expressed over platelet-derived growth factor receptor α (PDGFRα) in quiescent muscle progenitors and myoblasts using the C2C12 mouse myoblast cell line (43, 44). Moreover, activation of PDGFRβ can induce signaling through the ERK and PI3K/AKT pathways, promoting proliferative and migratory responses (36, 44). Notably, *Pdgfrb* expression is downregulated as progenitors differentiate, suggesting a temporally restricted role during early activation and expansion (44).

Adding further complexity, lineage-tracing experiments in a rotator cuff injury model demonstrated that *Pdgfrb* lineage cells can contribute to divergent outcomes (36). For instance, *Pdgfrb*^+^ cells that coexpress (*Pdgfra*) promote fibro-adipogenic remodeling and scar formation. Yet, a distinct PDGFRβ^+^ cell subset marked by SCA-1/CXCR4/integrin-β1 serves as muscle progenitors that directly contribute to regenerating myofibers (36). These findings highlight the heterogeneity of PDGFRβ lineage cells and raise the possibility that the context and signaling environment dictate whether PDGFRβ activity supports fibrosis, adipogenesis, or muscle regeneration (36). Although these prior studies have suggested roles for PDGFRβ in muscle progenitors, the specific contributions of PDGFRβ signaling to proliferation, differentiation, and fusion have not been fully clarified using muscle-specific genetic approaches (36, 45, 46).

In this study, we investigated the role of PDGFRβ signaling in skeletal muscle using genetic gain- and loss-of-function models and pharmacological modulation during myocyte differentiation and regeneration. Activation of *Pdgfrb* impaired myotube formation and myofiber regeneration independently of muscle cell renewal, whereas *Pdgfrb* deletion enhanced myonuclear accretion, fusion, and regeneration. Mechanistically, PDGFRβ signaling acted through STAT1 phosphorylation to modulate TGF-β and focal adhesion gene networks governing myocyte size and fusion. Pharmacological inhibition of PDGFRβ in murine and human models accelerated myofiber regeneration and myotube development, highlighting PDGFRβ as a target to enhance skeletal muscle repair.

## Results

### PDGFRβ signaling is active in muscle progenitor cells.

**Figure 1:**
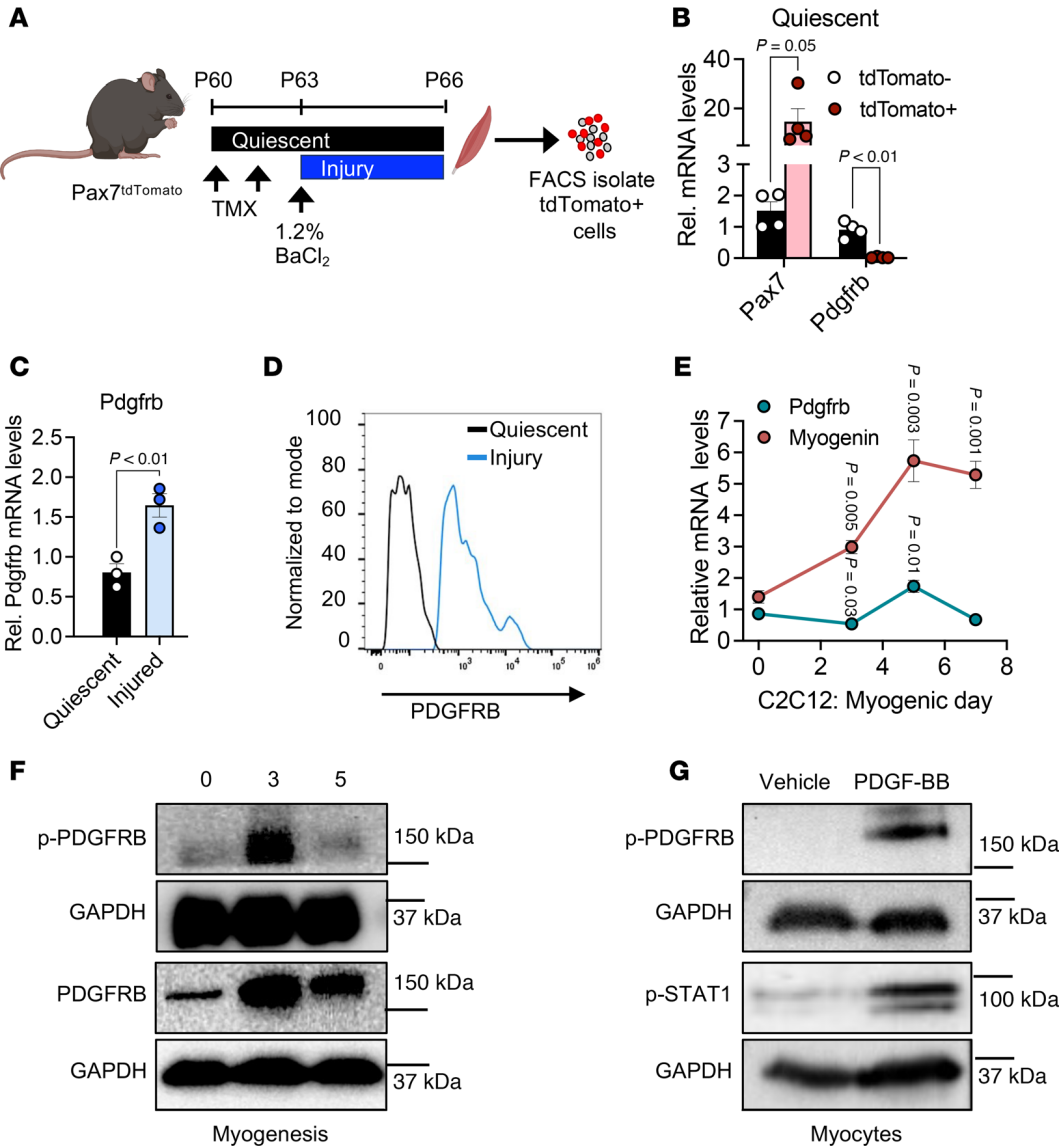
PDGFRβ is induced and activated in stimulated muscle progenitor cells. (**A**) Experimental design: Pax7^tdTomato^ male mice received TMX at P60 and were left uninjured or injured with 1.2% BaCl_2_. TA muscles were collected, dissociated, and Pax7^tdTomato^-positive and -negative cells were isolated by FACS. Panel A was created using BioRender. (**B**) qPCR analysis of *Pax7* and *Pdgfrb* in quiescent Pax7^tdTomato^-positive and -negative cells (*n* = 4 mice/group). (**C**) *Pdgfrb* mRNA levels in Pax7^tdTomato^ cells under quiescence or at 3 d.p.i. (*n* = 3 mice/group). (**D**) Flow cytometric histograms showing PDGFRβ surface expression on quiescent and injured Pax7^tdTomato^ cells. (**E**) Temporal *Pdgfrb* and *myogenin* mRNA expression during C2C12 differentiation (*n* = 4 independent cultures). (**F**) Immunoblot analysis of total and p-PDGFRβ levels during primary myogenic differentiation. (**G**) Immunoblot showing PDGFRβ and STAT1 phosphorylation in myocytes treated with vehicle or PDGF-BB (15 ng/mL, 15 min). Data represent the mean ± SEM. Statistical significance was determined using an unpaired, 2-tailed Student’s *t* test (**B**, **C**, and **E**). Rel., relative.

To define the expression pattern of *Pdgfrb* in muscle progenitors, we performed FACS to isolate quiescent PAX7^+^ cells from hind limb muscle groups using the tamoxifen-inducible (TMX-inducible) *Pax7-Cre^ERT2^* mouse model. To provide lineage visibility and isolation, we combined *Pax7-Cre^ERT2^* mice with the indelible genetic reporter *Rosa26^tdTomato^* (Pax7^tdTomato^) (Supplemental Figure 1A; supplemental material available online with this article; https://doi.org/10.1172/JCI188272DS1) (47, 48). We induced recombination by administering a single dose of TMX for 2 consecutive days, resulting in a high correspondence between reporter fluorescence and endogenous paired box protein 7 (PAX7) expression (Supplemental Figure 1, B–G). Mice were then randomized into uninjured (quiescent state) or chemically induced (1.2% BaCl_2_) injury groups. We then FACS-isolated Pax7^tdTomato^-positive and -negative cells at 0 and 3 days post injury (d.p.i.) (Figure 1A). *Pdgfrb* mRNA expression was barely detectable within the quiescent *Pax7*^+^ cells compared with the surrounding muscle stroma (Figure 1B). At 3 d.p.i., a time when MuSCs are activated (1), we found that *Pdgfrb* mRNA expression increased within *Pax7^+^* cells (Figure 1C). Consistent with these transcriptional changes, activated *Pax7^+^* cells expressed more PDGFRβ protein compared with quiescent MuSCs (Figure 1D). Notably, activated *Pax7^+^* cells lacked PDGFRα expression (43), indicating a selective PDGFRβ role in the muscle progenitor lineage (Supplemental Figure 1H).

To evaluate *Pdgfrb* expression across in vitro myogenesis, we differentiated C2C12 myoblasts (49, 50) and harvested RNA at various differentiation stages. We found that *Pdgfrb* expression tracked with *myogenin* but was rapidly downregulated upon myotube maturation, as previously observed (44) (Figure 1E). We confirmed by immunoblotting that total PDGFRβ and phosphorylated PDGFRβ (p-PDGFRβ) levels increased by day 3 and declined by day 5 during differentiation of primary myogenic progenitors (Figure 1F).

To determine whether PDGFRβ signaling can be activated during myogenesis, we differentiated primary progenitors for 3 days and stimulated them with PDGF-BB, a predominant PDGFRβ-activating ligand dimer (30), for 15 minutes. PDGF-BB induced robust PDGFRβ phosphorylation and activated downstream STAT1, a known effector of PDGFRβ signaling (51, 52) (Figure 1G). Overall, these data suggest that PDGFRβ is upregulated and activated during muscle progenitor differentiation.

### PDGFRβ activity alters myotube nuclear accretion.

**Figure 2:**
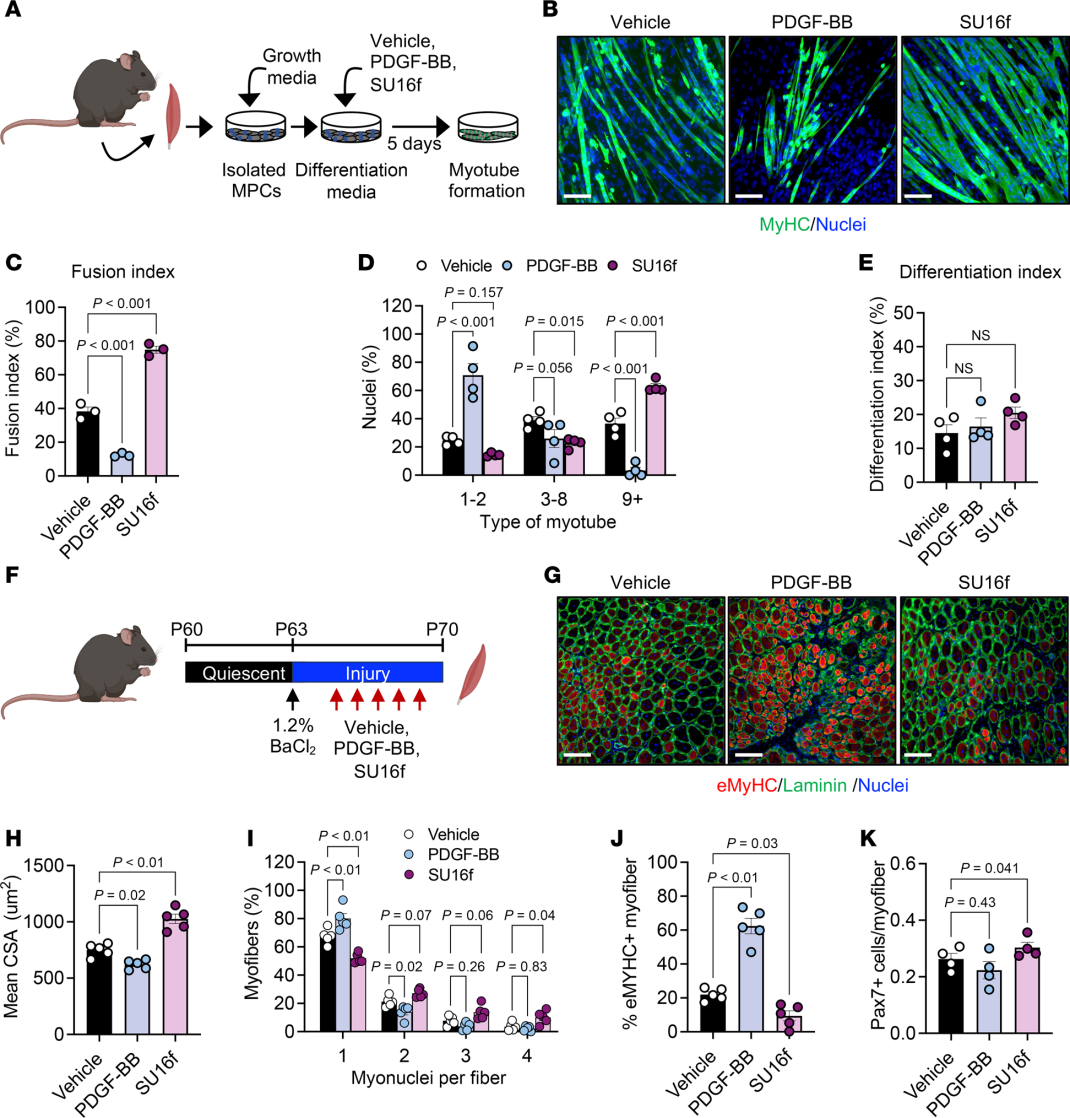
PDGFRβ activity regulates myotube development and muscle regeneration. (**A**) Experimental design: Muscle progenitor cells (MPCs) isolated from P30 male mice were differentiated and treated for 5 days with vehicle (0.1% DMSO), PDGF-BB (25 ng/mL), or SU16f (1 μM), and myotube formation was assessed. (**B**) Representative MyHC-stained images showing treatment-dependent differences in myotube formation. (**C**) Fusion index quantification from the cultures in **B** (*n* = 3 mice/group). (**D**) Quantification of myotube nuclear number and distribution from the cultures in **B** (*n* = 4 mice/group). (**E**) Differentiation index of low-density MyoG^tdTomato^ progenitors treated with vehicle, PDGF-BB, or SU16f (*n* = 4 mice/group). (**F**) In vivo regeneration protocol: MyoG^tdTomato^ mice received BaCl_2_ injury (1.2%), followed by daily injections of vehicle, PDGF-BB (50 ng/mouse), or SU16f (2 mg/kg) for 5 days. TA muscles were analyzed at 7 d.p.i. (**G**) Representative laminin and eMyHC immunostaining shows regeneration across treatment groups. (**H**) Quantification of injured myofiber CSA from the images in **G** (*n* = 5 mice/group). (**I**) Myonuclear numbers per injured myofiber from the images in **G** (*n* = 5 mice/group). (**J**) Quantification of eMyHC^+^ myofibers from the images in **G** (*n* = 5 mice/group). (**K**) Quantification of PAX7^+^ cells in regenerating TA muscle following the treatments described in **F** (*n* = 4 mice/group). Data represent the mean ± SEM. Statistical significance was determined by 1-way ANOVA (**C**, **E**, **H**, **J**, and **K**) or 2-way ANOVA (**D** and **I**) followed by multiple-comparison tests. Scale bars: 100 μm (**B** and **G**). Panels A and F were created using BioRender.

To determine whether PDGFRβ has a functional role in myotube development, we used in vitro PDGFRβ activation and inhibition strategies. Primary muscle progenitor cells isolated from hind limb muscles of male C57BL/6J-129SV mice (53–55) were differentiated and treated daily for 5 days with vehicle, PDGF-BB (25 ng/mL), or SU16f (1 μM), a potent and selective PDGFRβ inhibitor (51, 56) (Figure 2A). PDGFRβ inactivation by SU16f significantly increased multinucleated myotubes and the nuclear fusion index. Moreover, we observed increases in myotube length and diameter (Figure 2, B–D, and Supplemental Figure 2, A and B). In contrast, PDGF-BB treatment suppressed myotube formation, primarily producing small, nascent myotubes containing only 1–2 nuclei (Figure 2, B–D, and Supplemental Figure 2, A and B).

A potential confounder of PDGFRβ signaling could be altered myoblast differentiation, unrelated to myotube appearance (44). To assess this notion, we used the *myogenin*-*Cre* (MyoG) (57) mouse model combined with the *Rosa26^tdTomato^* model for myocyte labeling, facilitating the visualization of myocytes and scoring of myotube formation (Supplemental Figure 2C). We isolated muscle progenitors from the hind limb muscles of *MyoG^tdTomato^* mice. Critically, isolation of primary muscle cells from *MyoG^tdTomato^* mice was tdTomato^–^, confirming that *myogenin* was not actively expressed in freshly isolated muscle progenitors (Supplemental Figure 2D) (58). Conversely, when differentiated and fused, tdTomato was easily observed in myotubes and corresponded with the mature myotube marker myosin heavy chain (MyHC) (59) (Supplemental Figure 2E). Isolated muscle progenitors were cultured at low density and induced with ligands, along with differentiation media (26). We found that neither activation nor inhibition of PDGFRβ affected the formation of *MyoG^tdTomato^* myocytes, suggesting that PDGFRβ might not regulate myocyte differentiation (44) but rather myogenic fusion potential (Figure 2E and Supplemental Figure 2F). Notably, while MyHC staining remained comparable, cells treated with PDGF-BB remained rounded compared with the elongated morphology observed with SU16f treatment (Supplemental Figure 2G).

Consistent with our findings in primary muscle cell cultures, treatment of C2C12 cells with PDGF-BB markedly limited myotube formation (Supplemental Figure 3, A–C). Conversely, blocking PDGFRβ signaling significantly increased the number of multinucleated myotubes (Supplemental Figure 3, A–C). Since PDGF-BB can activate PDGFRα (39), we assessed whether chemical inhibition of PDGFRα alters in vitro myotube development. To this end, we isolated muscle progenitors from *Pax7^tdTomato^* mice and cultured them with TMX. Subsequently, muscle progenitors were treated with crenolanib, a selective PDGFRα inhibitor (60), at 10 and 100 nM throughout myotube development. However, under these conditions, we did not observe differences in myotube appearance, fusion index, nuclei accumulation, or differentiation index (Supplemental Figure 3, D–G). To further investigate the role of PDGFRβ in myotube formation, we conducted *Pdgfrb* shRNA–knockdown experiments in C2C12 cells. Like chemical inhibition, *Pdgfrb* knockdown led to a substantial increase in the number of nuclei within MyHC^+^ myotubes (Supplemental Figure 3, H and I). Overall, PDGFRβ activation appeared to impede myotube formation, while inhibition of PDGFRβ promoted myotube development and enhanced myonuclear accretion.

### Inhibition of PDGFRβ promotes myofiber regeneration.

The divergent effects of PDGFRβ activation and inhibition on in vitro myotube formation led us to test whether modulating this pathway influences myofiber regeneration in vivo. Tibialis anterior (TA) muscles were injured with 1.2% BaCl_2_, and mice were treated daily for 5 days with PDGF-BB (50 ng/mouse) or the PDGFRβ inhibitor SU16f (2 mg/kg), beginning 24 hours after injury (Figure 2F). Immunoblot analysis confirmed that SU16f effectively blocked PDGFRβ activation in differentiated myocytes (Supplemental Figure 4A). SU16f treatment enhanced regeneration, as shown by larger centrally nucleated myofibers and increased myonuclear accretion, consistent with the possibility of augmented fusion (Figure 2, G–I). SU16f also reduced the proportion of embryonic MyHC^+^ (eMyHC^+^) fibers, indicating more advanced repair after injury (59, 61) (Figure 2J). In contrast, PDGF-BB impaired regeneration, as evidenced by the development of smaller, disorganized, centrally nucleated myofibers with fewer nuclei and an increased abundance of eMyHC^+^ fibers, consistent with delayed repair (Figure 2, G–J). Notably, neither treatment altered PAX7^+^ cell numbers in injured TA muscles (Figure 2K and Supplemental Figure 4B). Collectively, these findings demonstrate that inhibition of PDGFRβ promoted myotube formation and accelerated myofiber regeneration, whereas PDGFRβ activation restricted fusion events and delayed repair.

### Modulating Pdgfrb expression alters skeletal muscle regeneration.

**Figure 3:**
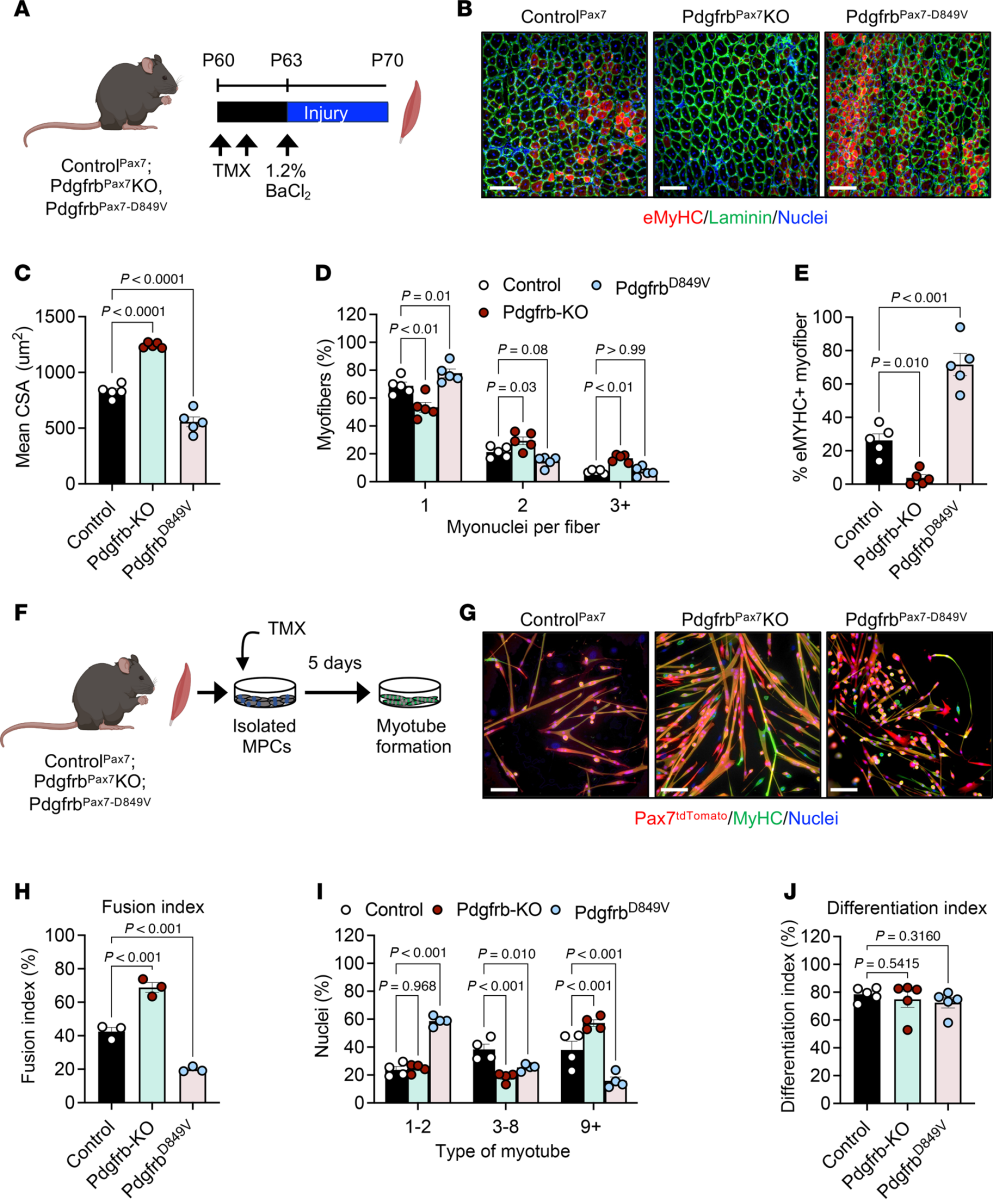
Genetic alteration of *Pdgfrb* expression changes muscle regeneration and myotube development. (**A**) Experimental design: Control^Pax7^, Pdgfrb^Pax7^-KO, and Pdgfrb^Pax7-D849V^ mice received TMX at P60 and subsequently injured with 1.2% BaCl_2_. TA muscles were analyzed at 7 d.p.i. (**B**) Representative laminin and eMyHC staining of injured TA sections from the mice described in **A**. (**C**) Mean CSA of injured myofibers from images described in **B** (*n* = 5 mice/group). (**D**) Myonuclear number per injured myofiber from the images described in **B** (*n* = 5 mice/group). (**E**) Quantification of eMyHC^+^ myofibers from the images described in **B** (*n* = 5 mice/group). (**F**) In vitro design: Muscle progenitors isolated from Control^Pax7^, Pdgfrb^Pax7^-KO, and Pdgfrb^Pax7-D849V^ mice were cultured with TMX, expanded, and differentiated to assess myotube development. (**G**) Representative Pax7^tdTomato^ and MyHC immunofluorescence images showing myotube formation from cultures in **F**. (**H**) Fusion index quantification from cultures in **G** (*n* = 3 mice/group). (**I**) Myotube nuclear number and distribution from cultures in **G** (*n* = 4 mice/group). (**J**) Differentiation index of low-density cultures from Control^Pax7^, Pdgfrb^Pax7^-KO, and Pdgfrb^Pax7-D849V^ mice, calculated from Pax7^tdTomato^ and myogenin colocalization (*n* = 5 mice/group). Data represent the mean ± SEM. Statistical significance was determined using 1-way ANOVA (**C**, **E**, **H**, and **J**) or 2-way ANOVA (**D** and **I**) followed by Dunnett’s multiple-comparison test (**D** and **I**). Scale bars: 100 μm (**B** and **G**). Panels A and F were created using BioRender.

Because pharmacologic PDGFRβ modulation may have off-target systemic effects, we next assessed the cell-autonomous role of PDGFRβ in muscle progenitors during regeneration. We generated Pdgfrb^Pax7^-KO mice by crossing *Pdgfrb^fl/fl^* (31) mice with Pax7-Cre^ERT2^ animals and incorporated the *Rosa26^tdTomato^* reporter for lineage tracing (Supplemental Figure 5A). Recombination was induced with 2 consecutive doses of TMX, and controls carrying the relevant alleles were treated identically (Figure 3A). Efficient loss of PDGFRβ signaling was confirmed by reduced PDGFRβ phosphorylation in isolated cells (Supplemental Figure 5, B and C). Adult mice were then subjected to TA injury using 1.2% BaCl_2_ and analyzed at 7 d.p.i. (Figure 3A). Under resting conditions, myofiber size was similar between genotypes (Supplemental Figure 5, D–F). Following injury, however, *Pdgfrb* deletion significantly increased the CSA of regenerating myofibers and enhanced myonuclear accretion, highlighting changes in fusion potential (Figure 3, B–D). Consistent with improved repair, eMyHC^+^ myofibers were largely absent in Pdgfrb^Pax7^-KO muscles (Figure 3, B and E). Notably, female mutant mice displayed comparable regenerative enhancement (Supplemental Figure 5, G–I).

To determine whether elevated PDGFRβ signaling impairs regeneration, we used an inducible gain-of-function model (Pdgfrb^Pax7-D849V^), which harbors a ligand-independent activating mutation in the PDGFRβ kinase domain (62) (Supplemental Figure 5A). We administered TMX to induce receptor activation in adult mice (Supplemental Figure 5, B and C). As with the KO model, baseline myofiber size did not differ between groups (Supplemental Figure 5, D–F). However, at 7 d.p.i., male Pdgfrb^Pax7-D849V^ muscles exhibited pronounced architectural disruption, reduced regenerating myofiber CSA, and fewer nuclei per fiber, indicating impaired fusogenic potential (Figure 3, B–D). Regeneration delay was further supported by elevated eMyHC immunostaining in *Pdgfrb*-activated muscles (Figure 3, B and E, and Supplemental Figure 5I). Notably, female *Pdgfrb*-activated mice showed similar trends (Supplemental Figure 5, G–I). These data collectively suggest that deletion of *Pdgfrb* in muscle progenitors facilitates myofiber growth, whereas activation of *Pdgfrb* hinders myofiber regeneration.

### Modulating Pdgfrb expression alters myotube development.

To determine whether PDGFRβ directly regulates myotube formation, we isolated muscle progenitors from control, Pdgfrb^Pax7^-KO, and Pdgfrb^Pax7-D849V^ mice, induced recombination with TMX in culture, and differentiated the cells into myotubes (Figure 3F). Deletion of *Pdgfrb* markedly increased the fusion index and the number of nuclei per myotube, indicating enhanced myonuclear accretion (Figure 3, G–I). In contrast, constitutive activation of *Pdgfrb* impaired myotube development, reducing the fusion index and yielding predominantly mono- and binucleated Pax7^tdTomato^ cells (Figure 3, G and H). Quantification confirmed that most Pdgfrb^Pax7-D849V^ cells remained in nascent myotubes and rarely formed multinucleated structures containing 9 or more nuclei (Figure 3I). Together, these findings demonstrate that *Pdgfrb* loss promoted myonuclear accretion and myotube growth, whereas *Pdgfrb* activation suppressed fusion and myotube maturation.

### PDGFRβ activity does not affect muscle progenitor cell proliferation.

To determine whether PDGFRβ influences muscle progenitor abundance during regeneration, we quantified Pax7^tdTomato+^ cells from TMX-induced control, Pdgfrb^Pax7^-KO, and Pdgfrb^Pax7-D849V^ mice at baseline and at 3 and 7 d.p.i. Flow cytometric analysis revealed no differences in Pax7^tdTomato+^ cell numbers across genotypes at any time point (Supplemental Figure 6A). Consistently, PAX7 immunostaining of injured muscle sections showed comparable PAX7^+^ cell abundance regardless of *Pdgfrb* loss or activation (Supplemental Figure 6, B and C). To assess muscle progenitor proliferation, at 3 d.p.i., we administered a single 5-ethynyl-2′-deoxyuridine (EdU) pulse. Using flow cytometry, we found that EdU incorporation in muscle progenitors was similar across genotypes (Supplemental Figure 6D). Because PDGFRα can compensate for PDGFRβ in other lineages, we also examined PDGFRα expression but found that Pax7^tdTomato+^ cells were uniformly PDGFRα^–^, independent of genotype (Supplemental Figure 6E).

We next asked whether PDGFRβ modulates myocyte differentiation. Muscle progenitors from control and mutant mice were isolated, induced with TMX, plated at low density, and differentiated (63) (Supplemental Figure 6F). The differentiation index, assessed by myogenin and MyHC expression within Pax7^tdTomato+^ cells, was comparable across genotypes (Figure 3J and Supplemental Figure 6, G and H). Altogether, these findings indicate that PDGFRβ signaling did not alter muscle progenitor numbers, proliferation, or differentiation, suggesting that PDGFRβ activity may regulate myocyte fusion.

### Reinjury model reveals consistent effects of Pdgfrb expression on regeneration.

Because we observed comparable numbers of Pax7^+^ cells in control, Pdgfrb^Pax7^-KO, and Pdgfrb^Pax7-D849V^ mice during the initial regeneration phase (Supplemental Figure 6, A–D), we next evaluated whether differences in myofiber growth persisted at later time points. TA muscles were harvested 28 days after injury to assess recovery (Supplemental Figure 7A). At this stage, myofibers from Pdgfrb^Pax7^-KO mice remained larger compared with controls, whereas myofiber CSAs from Pdgfrb^Pax7-D849V^ mice trended smaller (Supplemental Figure 7, B and C). Despite these differences in fiber size, Pax7^+^ cell abundance was similar across all genotypes (Supplemental Figure 7D).

To test whether these functional effects would persist under repeated stress, TA muscles were reinjured at 28 days and analyzed at 7 d.p.i (Supplemental Figure 7E). Consistent with the first injury model, deletion of *Pdgfrb* in PAX7^+^ progenitors enhanced regeneration, yielding larger regenerating fibers with increased nuclear accretion (Supplemental Figure 7, F–H). In contrast, constitutive activation of *Pdgfrb* expression impaired myofiber regeneration (Supplemental Figure 7, F–H). Together, these findings demonstrate that modulating *Pdgfrb* expression has durable effects on skeletal muscle regenerative capacity, both after initial regeneration and after repeated injury.

### Myocytes with altered Pdgfrb expression have different myofiber-regenerative outcomes.

**Figure 4:**
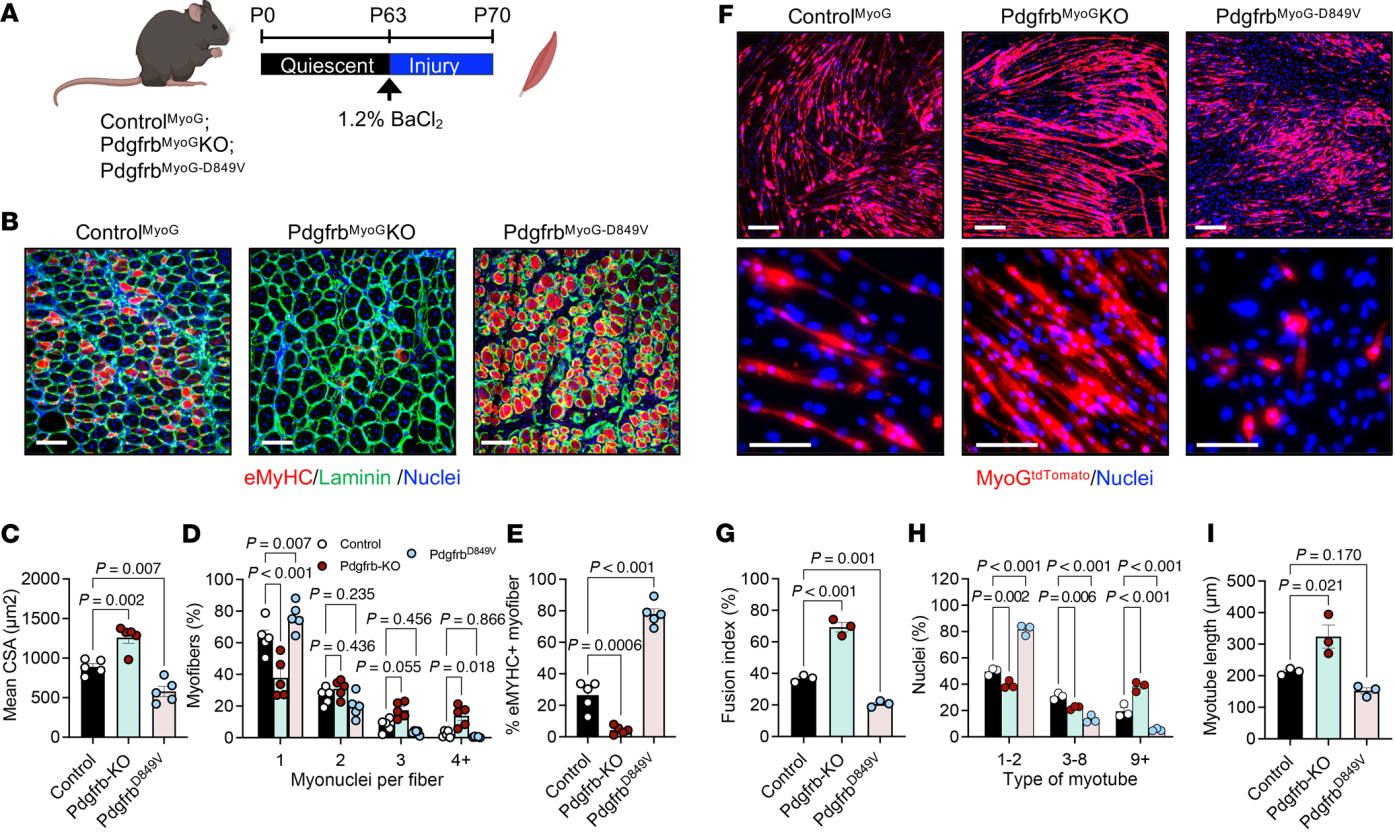
Genetically altering *Pdgfrb* activity in myocytes modifies muscle regeneration. (**A**) Experimental design: Control^MyoG^, Pdgfrb^MyoG^-KO, and Pdgfrb^MyoG-D849V^ mice underwent BaCl_2_ injury (1.2%) to the TA, and muscles were analyzed at 7 d.p.i. Panel A was created using BioRender. (**B**) Representative laminin and eMyHC staining of injured TA sections from mice described in **A**. (**C**) Mean CSA of injured myofibers from images described in **B** (*n* = 5 mice/group). (**D**) Myonuclear number per injured myofiber from images described in **B** (*n* = 5 mice/group). (**E**) Quantification of eMyHC^+^ myofibers from the images described in **B** (*n* = 5 mice/group). (**F**) In vitro assay: progenitor cells isolated from Control^MyoG^, Pdgfrb^MyoG^-KO, and Pdgfrb^MyoG-D849V^ hind limb muscles were expanded, differentiated, and assessed for myotube formation. Representative MyoG^-tdTomato^ fluorescence images of myotube formation. Original magnification, ×10 (top) and ×40 (bottom). (**G**) Fusion index quantification from the cultures in **F** (*n* = 3 mice/group). (**H**) Myotube nuclear number and distribution from the cultures in **F** (*n* = 3 mice/group). (**I**) Myotube length measurements from cultures in **F**, indicating maturation and growth (*n* = 3 mice/group). Data represent the mean ± SEM. Statistical significance was determined using 1-way ANOVA followed by Dunnett’s multiple-comparison test (**C**–**E** and **G**–**I**). (**B** and **F**) Scale bars: 100 μm.

Our cellular data indicated that PDGFRβ signaling acted within myocytes rather than MuSCs; therefore, we next tested the necessity and sufficiency of *Pdgfrb* within myocytes during regeneration. To do this, we used the *MyoG-Cre* mouse model in combination with *Pdgfrb*^fl/fl^ (Pdgfrb^MyoG^-KO) mice or *Pdgfrb^D849V^* (Pdgfrb^MyoG-D849V^) constitutively activated mice. Adult mice (P60) underwent TA injury, and regeneration was evaluated at 7 d.p.i. (Figure 4A). Deletion of *Pdgfrb* significantly enhanced regeneration, producing larger centrally nucleated myofibers with increased myonuclear accretion (Figure 4, B–D). In contrast, constitutive activation of *Pdgfrb* impaired repair; that is, Pdgfrb^MyoG-D849V^ muscles exhibited smaller regenerating myofibers and markedly reduced nuclear content — often only 1 nucleus per myofiber (Figure 4, B–D). Reduced immunostaining for eMyHC indicated enhanced myofiber regeneration in Pdgfrb^MyoG^-KO mice compared with controls (Figure 4, B and E). In contrast, a higher number of eMyHC^+^ myofibers were observed in Pdgfrb^MyoG-D849V^ mice, suggesting delayed regeneration (Figure 4, B and E).

Because myocyte-specific *Pdgfrb* deletion improved regeneration at 7 d.p.i., we next examined whether these effects were evident earlier in the repair process. TA muscles from Pdgfrb^MyoG^-KO and Pdgfrb^MyoG-D849V^ mice were analyzed at 5 d.p.i., a stage when progenitors are actively proliferating, differentiating, and initiating fusion (8) (Supplemental Figure 8A). At this early time point, Pdgfrb^MyoG^-KO myofibers exhibited CSAs comparable to those of control myofibers but had reduced eMyHC staining and increased myonuclear accretion, consistent with accelerated maturation (Supplemental Figure 8, B–E). In contrast, Pdgfrb^MyoG-D849V^ muscles exhibited smaller regenerating myofibers along with a trend toward increased eMyHC^+^ fibers (Supplemental Figure 8, B–E).

### Fusion defects in adult myocytes with altered PDGFRβ activity.

We next asked whether PDGFRβ signaling within myocytes regulates the formation of multinucleated myotubes in vitro. Primary muscle cells were isolated from control, Pdgfrb^MyoG^-KO, and Pdgfrb^MyoG-D849V^ mice and differentiated for 5 days. In this model, muscle progenitors are WT for *Pdgfrb* until myogenin expression, ensuring that PDGFRβ deletion or activation occurs only at the onset of myocyte differentiation (Supplemental Figure 2, C–E). Pdgfrb^MyoG^-KO cultures displayed robust myotube formation, whereas constitutive activation of *Pdgfrb* markedly impaired myotube development (Figure 4F). Quantification confirmed that Pdgfrb^MyoG^-KO cells exhibited the highest fusion index, while Pdgfrb^MyoG-D849V^ cultures showed minimal fusion (Figure 4, F and G). Nuclei count further demonstrated that *Pdgfrb* deletion promoted the formation of multinucleated myotubes, whereas *Pdgfrb* activation resulted in predominantly mononuclear cells (Figure 4, F–H). Consistent with enhanced fusion, Pdgfrb^MyoG^-KO myotubes were significantly longer, while Pdgfrb^MyoG-D849V^ myotubes tended to be stunted (Figure 4I). Taken together, the data suggest that PDGFRβ signaling may alter fusion potential.

### Fusion defects in Pdgfrb^-D849V^ myocytes are reversible.

**Figure 5:**
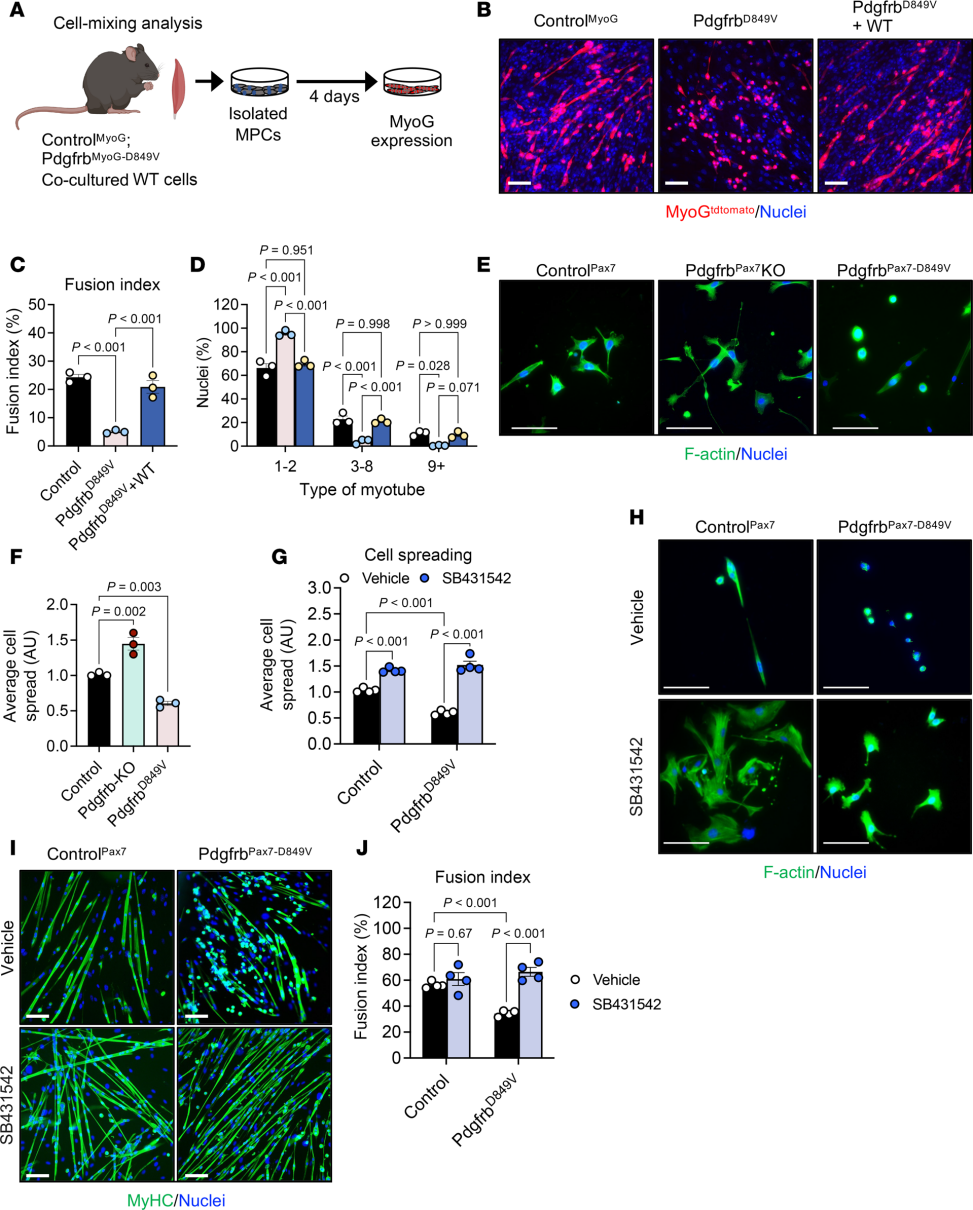
PDGFRβ signaling cooperates with TGF-β signaling to control myocyte morphology. (**A**) Experimental design for the in vitro cell-mixing experimental procedure: Muscle progenitor cells from Control^MyoG^, Pdgfrb^MyoG-D849V^, and WT mice were cultured either separately or as mixed populations, and myotube/chimeric myotube formation was assessed by MyoG^tdTomato^ fluorescence. Panel A was created using BioRender. (**B**) Representative MyoG^tdTomato^ images showing myotube development in the cultures described in **A**. (**C**) Fusion index quantification from the cultures in **B** (*n* = 3 mice/group). (**D**) Quantification of nuclei per myotube from the cultures in **B** (*n* = 3 mice/group). (**E**) Representative F-actin staining of myocyte morphology isolated from Control^Pax7^, Pdgfrb^Pax7^-KO, and Pdgfrb^Pax7-D849V^ mice. (**F**) Quantification of myocyte spreading from the cultures in **E** (*n* = 3 mice/group). (**G**) Quantification of myocyte spreading from the cultures in **H**, assessing PDGFRβ activity and TGF-β pathway inhibition (*n* = 4 mice/group). (**H**) Representative F-actin staining of Control^Pax7^ and Pdgfrb^Pax7-D849V^ myocytes treated with vehicle or SB431542 (5 μM). (**I**) Representative MyHC-stained images showing myotube formation in Control^Pax7^ and Pdgfrb^Pax7-D849V^ progenitor cells treated with vehicle or SB431542. (**J**) Fusion index quantification corresponding to the cultures shown in **I** (*n* = 4 mice/group). Data represent the mean ± SEM. Statistical significance was determined using 1-way ANOVA (**C** and **F**) or 2-way ANOVA (**D**, **G**, and **J**) followed by Šídák’s, Tukey’s, or Dunnett’s multiple-comparison test. Scale bars: 100 μm.

The inability of Pdgfrb^MyoG-D849V^ myocytes to form myotubes suggested a defect in the early steps required for fusion. To determine whether these cells could still fuse once the appropriate conditions were provided, we performed a cell-mixing assay (22) in which Pdgfrb^MyoG-D849V^ myocytes (tdTomato^+^) were cocultured with WT myocytes lacking MyoG^Cre^ and tdTomato expression (Figure 5A). As expected, control MyoG^tdTomato^ cells readily formed multinucleated myotubes, whereas Pdgfrb^MyoG-D849V^ cells in monoculture remained largely mononuclear with minimal myotube development (Figure 5B). When mixed with WT cells, Pdgfrb^MyoG-D849V^ myocytes successfully incorporated into developing myotubes, producing chimeric fibers with diffuse tdTomato labeling (Figure 5B). This rescue was reflected by a significant increase in the fusion index and a greater number of multinucleated myotubes (Figure 5, C and D). These results indicate that Pdgfrb^MyoG-D849V^ myocytes retained the capacity to fuse but were impaired in the preparatory cellular events that enable fusion to initiate.

### PDGFRβ signaling alters focal adhesion and TGF-β signaling pathways.

Culture studies revealed genotype-specific differences in myocyte morphology. Pdgfrb^MyoG-D849V^ cells became rounded, whereas Pdgfrb^MyoG^-KO cells were elongated, consistent with altered cytoskeleton remodeling (Figure 3G). Changes in actin remodeling have been shown to facilitate the creation of fusion protrusions that allow myocytes to interact, promoting cooperation between fusogenic proteins (26, 64, 65). Therefore, we hypothesized that PDGFRβ signaling may influence early morphological events that prepare cells for fusion. To test this, we plated TMX-induced Pdgfrb^Pax7^-KO and Pdgfrb^Pax7-D849V^ cells at low density and stained them for F-actin (66). Deletion of *Pdgfrb* promoted cell spreading and the formation of broad contact surfaces, whereas constitutive activation of *Pdgfrb* left most cells rounded and poorly interactive (Figure 5, E and F).

Our data suggest that PDGFRβ-induced cytoskeletal changes may be a critical determinant of altered fusion. Interestingly, cytoskeletal remodeling by TGF-β signaling has been shown to block cellular fusion (67). Likewise, PDGF and TGF-β signaling converge to regulate cytoskeletal dynamics in stromal fibroblasts (28), raising the possibility that PDGFRβ–TGF-β crosstalk influences myocyte fusion. To explore this connection, we treated control and Pdgfrb^Pax7-D849V^ myocytes with the TGF-β inhibitor SB431542 (5 μM) (68). Blocking TGF-β signaling restored spreading in Pdgfrb^Pax7-D849V^ cells and enabled them to establish contacts (Figure 5, G and H). When SB431542 was applied throughout differentiation, it restored the appearance of developing myotubes and significantly increased fusion and multinucleation in Pdgfrb^Pax7-D849V^ cultures (Figure 5, I and J, and Supplemental Figure 9A), without altering progenitor differentiation (Supplemental Figure 9B). Overall, these data suggest a possible cooperation between PDGFRβ and TGF-β signaling pathways to control cell morphology, throttling fusion events.

### PDGFRβ/STAT1 signaling regulates myogenic fusion.

To identify downstream mediators by which PDGFRβ activation disrupts myocyte spreading and fusion, we examined STAT1, a canonical PDGFRβ effector implicated in fibrosis, inflammatory signaling, and metabolic remodeling (51, 52, 62, 69). PDGF-BB stimulation induced rapid STAT1 phosphorylation in differentiated myocytes (Figure 1G and Supplemental Figure 4A), prompting us to test whether STAT1 activation is required for the fusion defects driven by PDGFRβ signaling. Treatment with fludarabine, an FDA-approved STAT1 inhibitor (70, 71), prevented PDGF-BB–induced STAT1 phosphorylation while leaving PDGFRβ phosphorylation intact (Supplemental Figure 10, A and B), enabling us to isolate STAT1-dependent signaling downstream of PDGFRβ.

**Figure 6:**
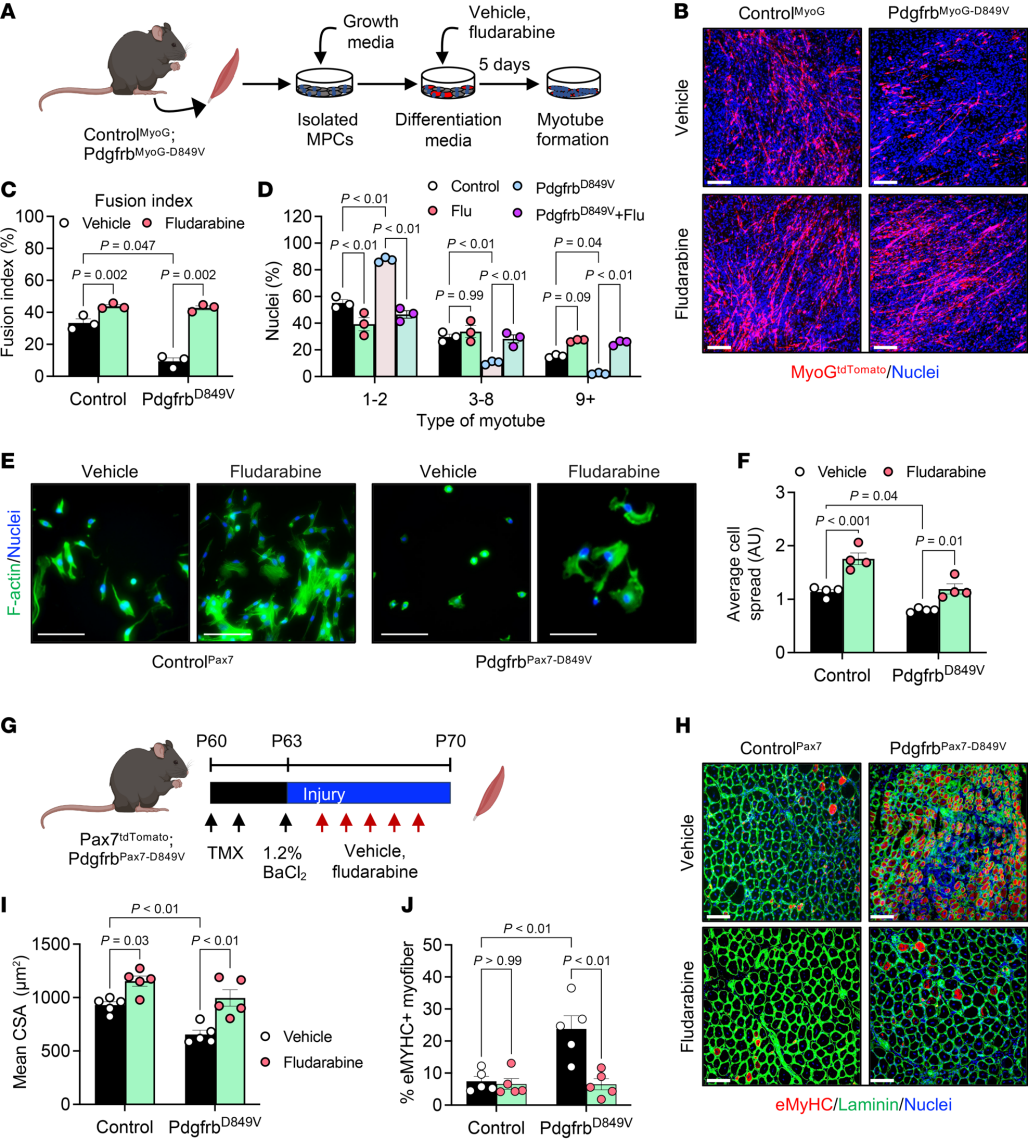
STAT1 mediates PDGFRβ signaling to control myocyte fusion and myofiber regeneration. (**A**) Experimental design for the in vitro assays. Muscle progenitor cells from Control^MyoG^ and Pdgfrb^MyoG-D849V^ mice were differentiated for 5 days in the presence of vehicle (0.1% DMSO) or fludarabine (1 μM), and myotube formation was assessed. (**B**) Representative MyoG^tdTomato^ images showing myotube development in the cultures described in **A**. (**C** and **D**) Fusion index (**C**) and nuclei per myotube (**D**) from the cultures described in **B** (*n* = 3 mice/group). (**E**) Representative F-actin staining of myocytes isolated from Control^MyoG^ and Pdgfrb^MyoG-D849V^ mice after 1 day of differentiation with vehicle or fludarabine (1 μM). (**F**) Quantification of cell spreading in the cultures shown in **E** (*n* = 4 mice/group). (**G**) In vivo design: Control^Pax7^ and Pdgfrb^Pax7-D849V^ mice received BaCl_2_ injury (1.2%) followed by daily injections of vehicle or fludarabine (3 mg/kg) for 5 days. TA muscles were analyzed at 7 d.p.i. (**H**) Representative laminin and eMyHC staining of injured TA sections from the mice described in **G**. (**I**) Mean CSA of injured myofibers from the images described in **H** (*n* = 5 mice/group). (**J**) Quantification of eMyHC^+^ myofibers from the images described in **H** (*n* = 5 mice/group). Data represent the mean ± SEM. Statistical significance was determined using 2-way ANOVA (**C**, **D**, **F**, **I**, and **J**) followed by Šídák’s or Tukey’s multiple-comparison test. Scale bars: 100 μm. Panels A and G were created using BioRender.

In C2C12 cultures, PDGF-BB reduced fusion and myonuclear accretion, whereas fludarabine alone enhanced both metrics. Importantly, fludarabine restored multinucleation in PDGF-BB–treated cultures, indicating that STAT1 activation was necessary for PDGFRβ-mediated suppression of fusion (Supplemental Figure 10, C–E). To test this in a more physiologic context, we treated primary myocytes from Control^MyoG^ and Pdgfrb^MyoG-D849V^ mice with fludarabine. Constitutive PDGFRβ activation severely restricted fusion, yet fludarabine markedly rescued myotube formation and myonuclear accumulation (Figure 6, A–D). A similar rescue was observed in Pdgfrb^Pax7-D849V^ cultures (Supplemental Figure 6, F–H), demonstrating that STAT1 inhibition overrode the fusion block imposed by hyperactive PDGFRβ signaling.

Because PDGFRβ activation also impaired early cytoskeletal remodeling, we asked whether STAT1 drives these morphological changes. Indeed, fludarabine increased spreading in control myocytes and restored F-actin organization and contact formation in Pdgfrb^Pax7-D849V^ cells (Figure 6, E and F), suggesting that STAT1 activation mediated *Pdgfrb* signaling to modulate cytoskeletal changes.

Finally, we tested whether inhibition of STAT1 improves muscle regeneration in vivo (Figure 6G). Following BaCl_2_ injury, Pdgfrb^Pax7-D849V^ mice displayed disrupted architecture, small regenerating fibers, and persistent eMyHC expression. Fludarabine treatment improved tissue organization, increased regenerating myofiber CSA, reduced eMyHC staining, and restored morphologic features of regular repair (Figure 6, H–J). Similar outcomes were observed in Pdgfrb^MyoG-D849V^ mice (Supplemental Figure 10I). Altogether, these data establish STAT1 as a critical downstream effector of PDGFRβ, facilitating cytoskeletal remodeling, myocyte spreading, and myofiber regeneration.

### PDGFRβ signaling regulates human muscle myotube formation.

**Figure 7:**
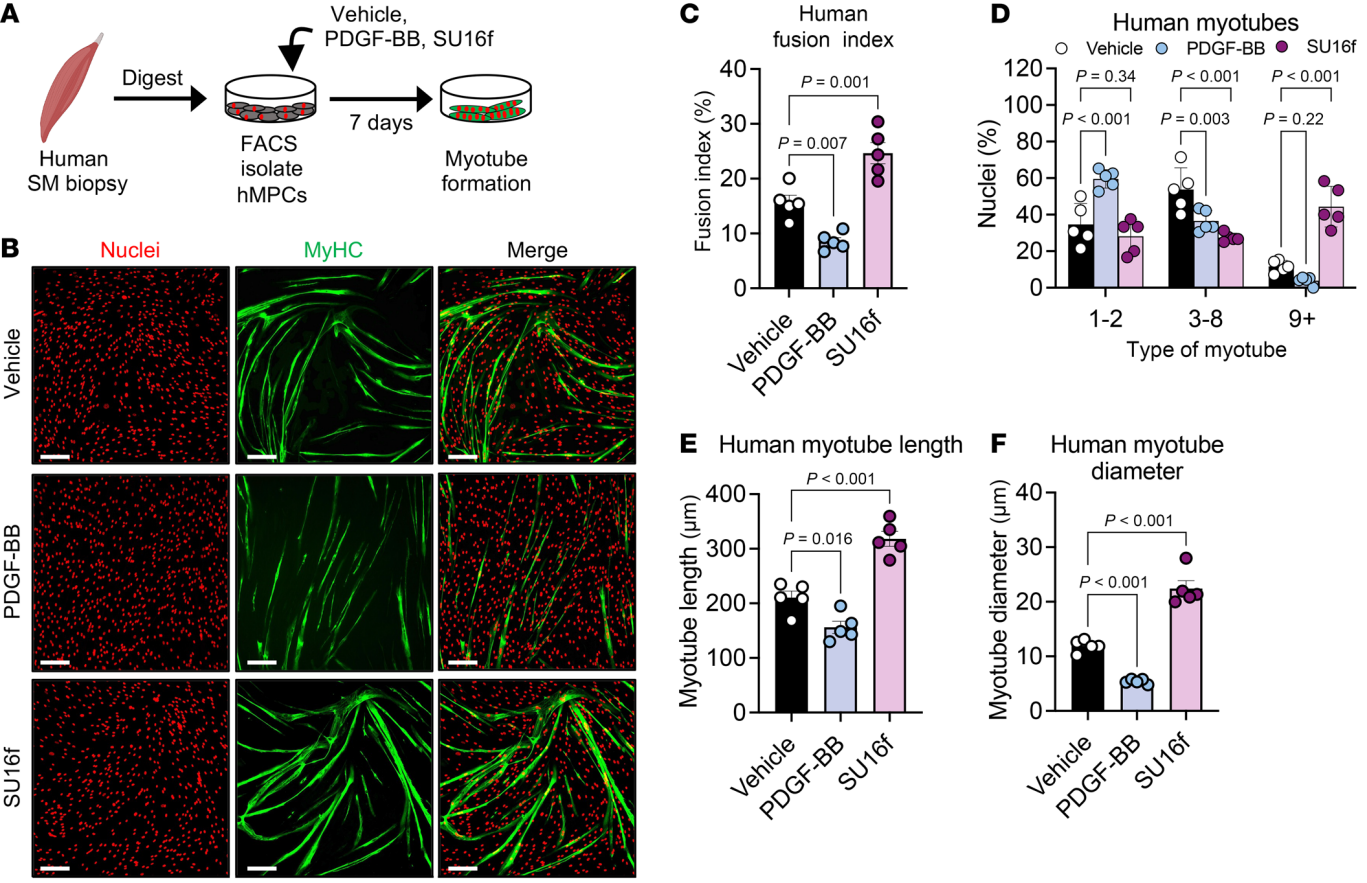
Blocking PDGFRβ signaling in human muscle cells boosts myotube development. (**A**) Experimental design: Human muscle progenitor cells were FACS-isolated from quadriceps biopsies, expanded, and differentiated in the presence of vehicle, PDGF-BB (25 ng/mL), or SU16f (1 μM). Myotube formation was assessed at the end of differentiation. Panel A was created using BioRender. (**B**) Representative MyHC-stained images showing treatment-dependent differences in myotube formation. Scale bars: 100 μm. (**C**–**F**) Quantification of the fusion index (**C**), nuclei per myotube (**D**), myotube length (**E**), and myotube diameter (**F**) from the cultures shown in **B** (*n* = 5 independent human samples/group). Data represent the mean ± SEM. Statistical significance was determined by 1-way ANOVA followed by Tukey’s or Dunnett’s multiple-comparison (**C**–**F**).

To assess the relevance of PDGFRβ signaling in human myogenesis, we tested whether pharmacologic modulation of PDGFRβ activity alters fusion in primary human muscle progenitors. To do so, we isolated crude muscle progenitors from human vastus lateralis biopsies obtained from young adult women. After 4 passages, we FACS-enriched for CD56^+^CD29^+^ muscle progenitors, expanded the sorted cells, and used them for downstream experiments (72) (Figure 7A and Supplemental Figure 11, A–C). Under serum-restricted differentiation, PDGF-BB markedly impaired MyHC^+^ myotube formation, reducing fusion index, myonuclear accretion, and the progression of nascent myotubes into mature syncytia (Figure 7, B–D). In contrast, inhibiting PDGFRβ with SU16f enhanced fusion, increased the number of multinucleated myotubes, and promoted greater myotube length and diameter (Figure 7, B–F). Consistent with a conserved PDGFRβ/STAT1 axis, fludarabine increased human myotube formation and myonuclear accretion, indicating that STAT1 contributed to the blockade imposed by PDGFRβ activation (Supplemental Figure 11, D–G). Together, these findings demonstrate that PDGFRβ activation suppressed, and its inhibition enhanced, human muscle cell fusion, revealing a conserved regulatory mechanism across species.

## Discussion

Skeletal muscle fusion is essential for muscle development and regeneration (3, 5, 8). While fusogenic proteins are necessary for mediating cell-to-cell fusion, the pathways that prime myocytes for fusion remain undefined. Here, we identify PDGFRβ as a negative regulator of myofiber repair by restricting cellular spreading and myocyte fusion. Our findings reveal that heightened PDGFRβ signaling impeded myocyte fusion by reducing cell spreading in conjunction with TGF-β signaling, thereby slowing the regenerative process. Notably, these observations resonate with human gain-of-function PDGFRβ variants, such as those found in Kosaki overgrowth syndrome and infantile myofibromas (73, 74), underscoring the importance of PDGFRβ hyperactivation in shaping progenitor behavior and tissue architecture at the organ-specific level. Consistent with the involvement of STAT1 in these conditions (52), we show that PDGFRβ/STAT1 signaling in myocytes constrained cytoskeletal remodeling, which suppressed fusion to delay regeneration. The activation of STAT1 downstream of PDGFRβ may signify a disrupted cellular state (51, 75), aligning with the well-established ability of PDGFRβ signaling to engage multiple kinase and transcription factor pathways that may influence various stages of muscle cell proliferation, differentiation, and fusion (32, 33).

PDGF/PDGFR signaling is known to promote progenitor proliferation and migration, particularly during embryogenesis and wound healing (29, 39, 42, 76). However, its direct role in myogenic commitment has been difficult to resolve due to its pleiotropic effects in vivo. For instance, PDGF-BB expands satellite cells in *Mdx* mice (41), but these effects may arise from changes in inflammation, fibrosis, or vascularization (77, 78). Similarly, tyrosine kinase inhibitors such as imatinib or nilotinib can modify myogenic behavior through multiple targets (79, 80). Moreover, in our study, we did not detect noticeable effects of PDGFRβ signaling on myocyte commitment, but instead observed changes in myotube development, myonuclear accretion, and myofiber regeneration, in alignment with changes in fusion. Although we evaluated regenerative capacity using myocyte-driven *Pdgfrb* (MyoG) genetic models, we did not examine whether developmental phenotypes could influence muscle composition, strength, or systemic metabolism (76). Furthermore, the upstream regulation of PDGFRβ within the muscle niche and its potential interactions with other growth factor pathways remain essential open questions for future study.

The timing of PDGFRβ activity further supports a transient regulatory role. For example, p-PDGFRβ peaks early after injury and diminishes as fusion progresses, suggesting that it may delay premature fusion until myocytes are prepared for incorporation into regenerating fibers (81). The upstream cues regulating PDGFRβ — ligand availability, stromal or endothelial sources of PDGFs, and temporal niche changes — remain critically unidentified. Our data also reveal functional crosstalk between the PDGFRβ/STAT1 and TGF-β pathways, both of which influence cytoskeletal remodeling (26, 28). Whether these pathways converge on shared transcriptional programs or act through parallel cytoskeletal effectors warrants future investigation.

Pharmacological inhibition of PDGFRβ in murine and human models enhances myotube development and myonuclear accretion, highlighting the potential of PDGFRβ-targeted therapies to improve muscle repair. In pathological conditions in which muscle regeneration is compromised, such as in muscular dystrophies or severe injuries (82, 83), fine-tuning the fusion process through PDGFRβ modulation could promote the formation of functional muscle tissue and mitigate aberrant repair. This concept paves the way for innovative interventions to optimize muscle regeneration by targeting specific phases of the regenerative process (84). Although systemic administration of SU16f or PDGF-BB may affect multiple niche cell types, our findings establish PDGFRβ as a central regulator of the cellular mechanics of fusion. By functioning as a molecular brake on cytoskeletal remodeling and fusion competence, PDGFRβ defines a tunable checkpoint in the regeneration program. Targeting this pathway represents a promising therapeutic strategy to enhance myocyte fusion and accelerate functional muscle repair.

## Methods

### Sex as a biological variable

Our study examined male and female animals, and similar findings are reported for both sexes. Human muscle biopsies were collected from female volunteers. It is unknown whether the findings are relevant for male individuals.

### Animals

*Pdgfrb^fl/fl^* (010977), Rosa26-tdTomato (007914), Pax7^CreERT2^ (017763), and Pdgfrb^D849V^ (018434) mice were purchased from The Jackson Laboratory. MyoG mice were provided by Eric Olson, Department of Molecular Biology, University of Texas Southwestern Medical Center, Dallas, Texas, USA. Myogenin^Cre^ or Pax7-Cre^ERT2^ mice were crossed with *Pdgfrb^fl/fl^* or Pdgfrb^D849V^ allele mice, and the resulting mice were subsequently crossed with Rosa26^-tdTomato^ for lineage tracing. All strains were maintained for 8 or more generations before experimentation. Mice were housed at approximately 23°C and approximately 35% humidity on a 14:10 light/dark cycle and were given chow (Teklad, LM-485) ad libitum. TMX (50 mg/kg; Cayman Chemicals) dissolved in sunflower oil (MilliporeSigma) was injected i.p. for 2 consecutive days to induce recombination. For pharmacologic studies, mice received daily i.p. injections of vehicle (0.1% DMSO), PDGF-BB (50 ng/mouse; Peprotech), SU16f (2 mg/kg; Tocris), or fludarabine (3 mg/kg; Tocris) for 5 days, starting 24 hours after injury. Male and female mice, aged P30–P60, were used in the experiments.

### Human MuSC culture

Human muscle progenitor cells were provided by Anna Thalacker-Mercer (Cornell University, Ithaca, New York, USA) (85). Healthy adults (21–40 years of age) were recruited for this study. Vastus lateralis biopsies were enzymatically dissociated, and cells were expanded in DMEM/F12 with 20% FBS and basic FGF (bFGF) (5 ng/mL). After 4 passages, MuSCs were FACS-enriched for CD54^+^CD29^+^ cells (BioLegend), replated on 1.4% rat collagen, expanded twice, and differentiated in DMEM/F12 with 5% horse serum and the indicated ligands. Myotube formation was assessed at day 7 (85).

### Skeletal muscle injury

Mice were anesthetized with 3% isoflurane, and hind limbs were shaved and sterilized with ethanol. Injury was induced by injecting 50 μL of 1.2% BaCl_2_ into the TA. Mice were euthanized 5 or 7 days after injury or at 28 d.p.i. for recovery studies. For reinjury, at 28 d.p.i., mice were anesthetized with 3% isoflurane, TA muscles were injected with 1.2% BaCl_2_, and muscles were harvested 7 days later.

### Histological analysis

The TA muscles were surgically removed and embedded horizontally in OCT compound (Tissue-Tek) using disposable embedding molds (Epredia Peel-A-Way), snap-frozen with liquid nitrogen, and stored at –80°C before sectioning.

### Immunofluorescence staining

Cryosections (20 μm) were cut on a Leica CM1950 and air-dried for 10 minutes at room temperature. OCT was removed with PBS, and hydrophobic barriers were drawn using a PAP pen (Vector Laboratories). Sections were fixed in 4% paraformaldehyde (Electron Microscopy Sciences, 15700) for 20 minutes, washed in staining buffer (1× PBS containing 1% FBS and 0.1% sodium azide), and quenched with 0.1 M glycine (PBS, pH 7.4) for 10 minutes. After two 5-minute washes, sections were permeabilized with 0.25% Triton X-100 in staining buffer for 10 minutes and blocked for 30 minutes in 5% BSA. Primary antibodies against laminin (1:200; MilliporeSigma L9393) and eMyHC (5 μg/mL; DSHB F1.652) were applied overnight at 4°C in staining buffer. Following 2 PBS washes, secondary antibodies (Invitrogen, Thermo Fisher Scientific; anti–mouse IgG1-647, anti–rabbit Alexa Fluor 488 or 526; 1:200) were applied for 2 hours at room temperature. Nuclei were stained with Hoechst 33342 (1 μg/mL) for 10 minutes, followed by 3 PBS washes and mounting with Immu-Mount (Thermo Fisher Scientific). Images were acquired on a Leica DMi8 microscope using identical exposure settings across conditions.

PAX7 staining: Sections were air-dried for 1 hour and fixed in 4% PFA for 7 minutes. After three 5-minute PBS washes, they were blocked in 3% BSA (1 hour), incubated with laminin (1:50) overnight, and washed. Sections were then treated with 1× R-Buffer A (Electron Microscopy Sciences) at 95°C for 11 minutes for antigen retrieval, cooled to room temperature, and washed. Slides were blocked sequentially in M.O.M. IgG block (Vector Laboratories) for 1 hour and in 10% goat serum for 1 hour. Anti-PAX7 antibody (2 μg/mL; Developmental Studies Hybridoma Bank [DSHB]) was applied for 1 hour at room temperature and incubated overnight at 4°C. After washing, sections were incubated with biotinylated goat anti–mouse secondary antibody (1:1,000) for 70 minutes, followed by HRP-streptavidin (Thermo Fisher Scientific, Tyramide SuperBoost kit) for 1 hour followed by an 8-minute reaction. The reaction was stopped with 100 μL stop buffer, and slides were washed, post-fixed in 4% PFA for 5 minutes, counterstained with Hoechst (1 μg/mL), and mounted. Fluorescence images were acquired on a Leica DMi8.

### Histological quantification

All quantification was performed using Fiji ImageJ (NIH), with investigators blinded to the genotype.

#### PAX7 number quantification.

TA sections were stained for PAX7, laminin, and Hoechst. All PAX7^+^ nuclei were manually counted and normalized to the total number of myofibers imaged per section.

#### eMyHC quantification.

eMyHC staining was performed on TA muscles, with 70% of the total muscle injured. The number of eMyHC^+^ fibers was counted and normalized to the total number of injured muscle fibers. The results are expressed as the percentage of eMyHC^+^ fibers out of the total number of injured fibers.

#### CSA quantification.

Myofiber CSA was determined by laminin staining and was manually circled in Fiji ImageJ. For injury studies, only injured fibers with centralized nuclei were quantified, whereas all myofibers were quantified in noninjured specimens. For nuclei per injured myofiber, the nuclei within injured myofibers were manually quantified and classified as having 1, 2, 3, or 4 centrally located nuclei, and then divided by the total number of injured fibers per image.

### Muscle stem cell isolation

Gastrocnemius, quadriceps, and hamstrings muscles were dissected, and the tissue was minced and digested at 37°C with collagenase D at 20 mg/mL (Roche, 11088882001) and dispase II at 8 U/mL (Roche, 04942078001) for 120 minutes with gentle rocking. Digests were filtered through 70 μm strainers, cells were pelleted at 500*g* for 6 minutes, and RBCs were lysed in 1× RBC lysis buffer (BioLegend 420301) for 5 minutes on ice. Quenching was performed with 5 mL DMEM/F12 with 10% FBS, followed by filtering through 40 μm strainers and another centrifugation at 500*g* for 6 minutes. For negative selection, cells were incubated for 20 minutes on ice in MACS buffer (HBSS, 2% FBS, 2 mM EDTA) containing biotinylated antibodies against CD45 (BioLegend, 103104), CD11b (BioLegend, 101204), CD31 (BioLegend, 102404), and Sca1 (BioLegend, 108104). Cells were then washed, pelleted, and incubated with streptavidin magnetic beads (Invitrogen, Thermo Fisher Scientific, MSNB-6002-74) for 10 minutes at room temperature. Unbound MuSCs were collected by placing the suspension on a magnet for 5 minutes, washed, and plated on non-collagen-coated plastic for 1 hour at 37 °C and 5% CO_2_, followed by collection of nonadherent MuSCs for use in experiments.

### Cell culture

Following isolation, MuSCs from control and mutant tdTomato^+^ mice were resuspended in growth medium (DMEM/F12, 10% FBS, 5 ng/mL bFGF; Peprotech 100-18B). Cells were counted by a hemocytometer and plated on collagen-coated plates (Corning, 354236) at 50,000 cells/well for expansion or 10,000 cells/well for low-density differentiation assays. Cultures were maintained at 37°C, 5% CO_2,_ and the growth media were replaced 72 hours after plating. For in vitro recombination, cells were treated daily with 1 μM TMX until confluent. Differentiation was induced by switching to DMEM/F12 containing 5% horse serum, with media replaced daily. Cells were differentiated for 5 days before fixation and immunostaining. For cell-mixing (chimera) experiments, Control^MyoG^, Pdgfrb^MyoG-D849V^, or WT MuSCs were isolated as described in Muscle stem cell isolation, above. Control^MyoG^ and Pdgfrb^MyoG-D849V^ MuSCs were plated at 50,000 cells/well. For mixed-genotype assays, Pdgfrb^MyoG-D849V^ and WT MuSCs were combined at equal densities (*n* = 25,000 cells of each genotype) and differentiated under identical conditions. For C2C12 myoblast (American Type Culture Collection [ATCC], CRL-1772) assays, cells were cultured in growth media (DMEM with 10% FBS) on uncoated plates and used up to passage 5. At approximately 70% confluence, cells were switched to differentiation media (DMEM with 2% horse serum) and maintained for 5 days with daily media changes. Cells were fixed in 4% PFA for 45 minutes at room temperature before immunostaining. Three *Pdgfrb* lentiviral shRNAs (RMMM3981-201787548; RMM3981-201795622; RMM3981-201794272; Dharmacon Reagents) were pooled and transfected into C2C12 using Lipofectamine 3000 (Invitrogen, Thermo Fisher Scientific). Alternatively, the GFP-shRNA positive control (RHS4459) was transfected. For all in vitro assays, cells were treated at the onset of differentiation with vehicle (0.1% DMSO), PDGF-BB (25 ng/mL; VWR (Avantor), 10780-774), SU16f (1 μM; Tocris 3304), SB431542 (5 μM; Tocris, 1614), or fludarabine (1 μM; Tocris, 3495), and treatments were refreshed daily with each media change until fixation.

### Immunostaining of cultured cells

After fixation, cells were washed 3 times for 5 minutes in 1× TBS, permeabilized in 0.3% Triton X-100 in TBS for 30 minutes, and blocked in 5% donkey serum in TBS for 30 minutes at room temperature. Cells were incubated overnight at 4°C with primary antibodies at 1:100 in blocking buffer (Myosin Heavy Chain, Invitrogen, Thermo Fisher Scientific, MYSN02; Myogenin F5D, eBioscience 14-5643-82). Subsequently, cells were washed 3 times in TBS and incubated with Cy5 donkey anti–mouse secondary antibody (Jackson ImmunoResearch, 715-175-150) at 1:100 for 2 hours at room temperature in the dark. Counterstaining was done with Hoechst (1:1,000 in TBS) for 10 minutes, followed by imaging on a Leica DMi8. Two-three fields per replicate (20×) were quantified with at least 3 biological replicates per condition.

### Image-based myogenesis quantification

The fusion index was calculated as the number of nuclei within MyHC^+^ or tdTomato^+^ myotubes containing 3 or more nuclei, divided by the total number of nuclei per image. The differentiation index was calculated using low-density plating, with the number of nuclei within MyHC^+^ or tdTomato^+^ cells (mononucleated and multinucleated) divided by the total number of nuclei. MyHC^+^ or tdTomato^+^ myotubes were classified according to the number of nuclei (1–2, 3–8, or ≥9). For myotubes with 3 or more nuclei, the thinnest, thickest, and a representative mid-segment were measured to calculate the myotube diameter. To calculate myotube length, the myotube length was measured to compute per-image averages.

### RNA isolation and quantitative PCR

We homogenized 1 TA per mouse in 1 mL TRIzol (Ambion, Thermo Fisher Scientific, 15596) with metal beads using a Precellys 24. For cells, TRIzol was added directly to dishes or pellets. RNA was extracted by chloroform separation and isopropanol precipitation. RNA pellets were washed twice with 70% ethanol, and RNA concentration was determined on a TECAN Infinite F-nano+. cDNA was synthesized from 1 μg RNA using the High-Capacity RNA-to-cDNA kit (Thermo Fisher Scientific, 4368813), followed by dilution of cDNA 1:10 and quantitative PCR (qPCR) analysis using PowerUp SYBR Green (Thermo Fisher Scientific, A25742) on a QuantStudio 3. Data were analyzed using the ΔΔCt method with Rn18s as the internal control, and 4 technical replicates were performed per biological sample. The primer sequences were as follows: *Rn18s* forward 5′-GTAACCCGTTGAACCCCATT-3′, reverse 5′-CCATCCAATCGGTAGTAGCG-3′; *Pdgfrb* forward 5′-AGGGGGCGTGATGACTAGG-3′, reverse 5′-TTCCAGGAGTGATACCAGCTT-3′; and *Pax7* forward 5′-TCTCCAAGATTCTGTGCCGAT-3′, reverse 5′-CGGGGTTCTCTCTCTTATACTCC-3′.

### Flow cytometry

MuSCs were suspended in FACS buffer (1× PBS with 2.5% FBS and 2 mM EDTA), filtered through 5 mL strainer-cap tubes (BD Falcon), and tdTomato^-^ and tdTomato^+^ cells were sorted on a BD FACSAria Fusion for gene expression analyses. For intracellular staining, cells were fixed in 4% PFA for 30 minutes at room temperature, permeabilized in 0.3% Triton X-100 in TBS for 30 minutes, and blocked in PBS with 3% BSA and 5% goat serum for 1 hour. Cells were incubated with nonconjugated Pax7 (DSHB) at 1:100 in blocking buffer overnight at 4°C, washed, and then stained with goat anti–mouse Alexa Fluor 488 (Invitrogen, Thermo Fisher Scientific, A-11001) at 1:100 for 2 hours at room temperature. For surface markers, cells were stained with conjugated antibodies for 30 minutes on ice using the following dilutions: CD140b/PDGFRβ Alexa 488 at 1:200 (Invitrogen, Thermo Fisher Scientific, 53-1402-82) and CD140a/PDGFRα FITC at 1:200 (Invitrogen, Thermo Fisher Scientific, 11-1401-82). Samples were analyzed on a Thermo Fisher Scientific Attune NxT, and data were processed in FlowJo.

### EdU incorporation studies

TA injury was induced with 1.2% BaCl_2_ as described above. At quiescence and at 3 dpi, EdU was administered i.p. at 100 mg/kg 8 hours before harvesting. Injured muscles were dissociated and MuSCs isolated as described above. EdU was measured using the Click-iT Plus EdU Alexa Fluor 647 Flow Cytometry Kit (Thermo C10634) following the manufacturer’s instructions, with excitation at 633 nm and detection with a 660/20 nm filter on an Attune NxT. Data analysis was performed in FlowJo, and the percentage of PAX7^+^EdU^+^ MuSCs was quantified.

### Immunoblotting

Primary MuSCs were plated in 10 cm dishes, grown to approximately 50% confluence, switched to differentiation media for 24 hours, and serum-starved for 6 hours. Cells were then stimulated with vehicle (0.1% DMSO) or 15 ng/mL PDGF-BB for 15 minutes. For fludarabine experiments, cells were treated with 1 μM fludarabine or vehicle for 24 hours, followed by 12 hours of serum-free starvation with the same treatments, and then stimulation with 15 ng/mL PDGF-BB or vehicle for 15 minutes. Cells were lysed on ice in 200 μL RIPA buffer supplemented with protease and phosphatase inhibitors for 30 minutes, the lysates were cleared at 12,500*g* for 15 minutes at 4°C, and protein was quantified with the Pierce BCA assay on a TECAN Infinite F-nano+. Protein (50 μg) was mixed with 6× SDS/DTT sample buffer at a 1:5 lysate/buffer ratio, heated at 100°C for 10 minutes, and resolved on 10% SDS-PAGE at 90 V for approximately 2 hours in 1× running buffer (Bio-Rad, 1610744). Proteins were transferred to Immobilon PSQ PVDF membranes (MilliporeSigma, ISEQ0005) for 1 hour at 100 V on ice in 1× transfer buffer (Bio-Rad, 1610771), blocked in 5% BSA in TBS-T for 1 hour, and incubated overnight at 4°C with primary antibodies diluted in 5% BSA/TBS-T: p-PDGFRβ (Y1009, 1:1,000; Cell Signaling Technology, 3124S), GAPDH (1:1,000; Cell Signaling Technology, 2118), and p-STAT1 (Tyr701, 1:1,000; Invitrogen, Thermo Fisher Scientific, 33-3400). After TBS-T washes, cells were incubated with HRP-linked secondary antibodies (Cell Signaling Technology, 7076S or 7074S) for 2 hours at room temperature, developed with SuperSignal West Pico PLUS (Thermo Fisher Scientific, 34580) for 3 minutes, and imaged on a ProteinSimple FluorChem E or a Bio-Rad ChemiDoc MP (catalog 12003154).

### Statistics

Statistical analyses were performed using GraphPad Prism (versions 7–9). Comparisons between 2 groups were made using an unpaired, 2-tailed Student’s *t* test. For experiments involving more than 2 groups or conditions,1- or 2-way ANOVA with the appropriate post hoc testing was applied. Data are presented as the mean ± SEM, with individual data points shown where possible. A *P* value of less than 0.05 was considered statistically significant. The specific statistical tests and *n* values of biological replicates are provided in the figure legends. All experiments were repeated at least twice with a minimum of 3 biological replicates per group. Image acquisition and analysis were performed using Leica Application Suite X and FlowJo (version 10.8.1). Figures were prepared in PowerPoint, with select elements created using BioRender (https://www.biorender.com/).

### Study approval

All animal procedures were conducted in compliance with Cornell University and the IACUC guidelines under protocol 2017-0063 and with NIH guidelines. Human muscle biopsies were obtained through the Human Metabolic Research Unit at Cornell University. The study was approved by the Cornell IRB (protocol IDs: 1407004819, 1508005758, and 1704007090), and all participants provided written informed consent in accordance with the Declaration of Helsinki.

### Data availability

Values for all data points in graphs are reported in the Supporting Data Values file.

## Author contributions

SX and AMB performed the experiments, collected data, and analyzed the results. JEB and ATM collected, expanded, and prepared human muscle cells. BDC provided assistance and consultation on study design and experimental methodologies. SX, AMB, and DCB conceived and designed the study and wrote the manuscript. Co–first author order was determined by mutual discussion and agreement.

## Funding support

This work is the result of NIH funding, in whole or in part, and is subject to the NIH Public Access Policy. Through acceptance of this federal funding, the NIH has been given a right to make the work publicly available in PubMed Central.

- National Institute of Diabetes and Digestive and Kidney Diseases (NIDDK), NIH (R01-DK132264, to DCB).
- Cornell University (institutional funds to DCB).

## Supplementary Material

Supplemental data

Unedited blot and gel images

Supporting data values

## Version 1 02/16/2026

Electronic publication

## References

[1] Yin H (2013). Satellite cells and the muscle stem cell niche. Physiol Rev.

[2] Mauro A (1961). Satellite cell of skeletal muscle fibers. J Biophys Biochem Cytol.

[3] Motohashi N, Asakura A (2014). Muscle satellite cell heterogeneity and self-renewal. Front Cell Dev Biol.

[4] Segales J (2016). Regulation of muscle stem cell functions: a focus on the p38 MAPK signaling pathway. Front Cell Dev Biol.

[5] Tedesco FS (2010). Repairing skeletal muscle: regenerative potential of skeletal muscle stem cells. J Clin Invest.

[6] Tidball JG (2011). Mechanisms of muscle injury, repair, and regeneration. Compr Physiol.

[7] Wosczyna MN, Rando TA (2018). A muscle stem cell support group: coordinated cellular responses in muscle regeneration. Dev Cell.

[8] Charge SB, Rudnicki MA (2004). Cellular and molecular regulation of muscle regeneration. Physiol Rev.

[9] Fukada SI (2018). The roles of muscle stem cells in muscle injury, atrophy and hypertrophy. J Biochem.

[10] Chen EH (2007). Cell-cell fusion. FEBS Lett.

[11] Kim JH, Chen EH (2019). The fusogenic synapse at a glance. J Cell Sci.

[12] Griffin CA (2009). MOR23 promotes muscle regeneration and regulates cell adhesion and migration. Dev Cell.

[13] Charrin S (2013). Normal muscle regeneration requires tight control of muscle cell fusion by tetraspanins CD9 and CD81. Nat Commun.

[14] Kaufmann U (1999). The M-cadherin catenin complex interacts with microtubules in skeletal muscle cells: implications for the fusion of myoblasts. J Cell Sci.

[15] Kim JH (2015). Mechanical tension drives cell membrane fusion. Dev Cell.

[16] Schwander M (2003). Beta1 integrins regulate myoblast fusion and sarcomere assembly. Dev Cell.

[17] Shilagardi K (2013). Actin-propelled invasive membrane protrusions promote fusogenic protein engagement during cell-cell fusion. Science.

[18] Doherty KR (2005). Normal myoblast fusion requires myoferlin. Development.

[19] Bi P (2017). Control of muscle formation by the fusogenic micropeptide myomixer. Science.

[20] Goh Q, Millay DP (2017). Requirement of myomaker-mediated stem cell fusion for skeletal muscle hypertrophy. Elife.

[21] Leikina E (2018). Myomaker and myomerger work independently to control distinct steps of membrane remodeling during myoblast fusion. Dev Cell.

[22] Millay DP (2013). Myomaker is a membrane activator of myoblast fusion and muscle formation. Nature.

[23] Millay DP (2014). Myomaker is essential for muscle regeneration. Genes Dev.

[24] Zhang Q (2017). The microprotein Minion controls cell fusion and muscle formation. Nat Commun.

[25] Sampath SC (2018). Myoblast fusion confusion: the resolution begins. Skelet Muscle.

[26] Girardi F (2021). TGFβ signaling curbs cell fusion and muscle regeneration. Nat Commun.

[27] Brack AS (2008). A temporal switch from notch to Wnt signaling in muscle stem cells is necessary for normal adult myogenesis. Cell Stem Cell.

[28] Contreras O (2019). Cross-talk between TGF-β and PDGFRα signaling pathways regulates the fate of stromal fibro-adipogenic progenitors. J Cell Sci.

[29] Armulik A (2011). Pericytes: developmental, physiological, and pathological perspectives, problems, and promises. Dev Cell.

[30] Heldin CH, Westermark B (1999). Mechanism of action and in vivo role of platelet-derived growth factor. Physiol Rev.

[31] Schmahl J (2008). The PDGF signaling pathway controls multiple steroid-producing lineages. Genes Dev.

[32] Tallquist M, Kazlauskas A (2004). PDGF signaling in cells and mice. Cytokine Growth Factor Rev.

[33] Tallquist MD (2003). Additive effects of PDGF receptor beta signaling pathways in vascular smooth muscle cell development. PLoS Biol.

[34] Kostallari E (2015). Pericytes in the myovascular niche promote post-natal myofiber growth and satellite cell quiescence. Development.

[35] Dellavalle A (2007). Pericytes of human skeletal muscle are myogenic precursors distinct from satellite cells. Nat Cell Biol.

[36] Dar A (2024). Lineage tracing reveals a novel PDGFRβ^+^ satellite cell subset that contributes to myo-regeneration of chronically injured rotator cuff muscle. Sci Rep.

[37] Lu A (2022). The role of the aging microenvironment on the fate of PDGFRβ lineage cells in skeletal muscle repair. Stem Cell Res Ther.

[38] Dellavalle A (2011). Pericytes resident in postnatal skeletal muscle differentiate into muscle fibres and generate satellite cells. Nat Commun.

[39] Yablonka-Reuveni Z (1990). Regulation of proliferation and differentiation of myoblasts derived from adult mouse skeletal muscle by specific isoforms of PDGF. J Cell Biol.

[40] Hamaguchi H (2023). PDGF-B secreted from skeletal muscle enhances myoblast proliferation and myotube maturation via activation of the PDGFR signaling cascade. Biochem Biophys Res Commun.

[41] Pinol-Jurado P (2017). Platelet-derived growth factor bb influences muscle regeneration in duchenne muscle dystrophy. Am J Pathol.

[42] Yablonka-Reuveni Z, Seifert RA (1993). Proliferation of chicken myoblasts is regulated by specific isoforms of platelet-derived growth factor: evidence for differences between myoblasts from mid and late stages of embryogenesis. Dev Biol.

[43] Uezumi A (2014). Identification and characterization of PDGFRα+ mesenchymal progenitors in human skeletal muscle. Cell Death Dis.

[44] Contreras O (2021). PDGF-PDGFR network differentially regulates the fate, migration, proliferation, and cell cycle progression of myogenic cells. Cell Signal.

[45] An X (2023). Inhibition of PDGFRβ alleviates endothelial cell apoptotic injury caused by DRP-1 overexpression and mitochondria fusion failure after mitophagy. Cell Death Dis.

[46] Gerli MFM (2019). Combined notch and PDGF signaling enhances migration and expression of stem cell markers while inducing perivascular cell features in muscle satellite cells. Stem Cell Reports.

[47] Murphy MM (2011). Satellite cells, connective tissue fibroblasts and their interactions are crucial for muscle regeneration. Development.

[48] Madisen L (2010). A robust and high-throughput Cre reporting and characterization system for the whole mouse brain. Nat Neurosci.

[49] Blau HM (1983). Cytoplasmic activation of human nuclear genes in stable heterocaryons. Cell.

[50] Yaffe D, Saxel O (1977). A myogenic cell line with altered serum requirements for differentiation. Differentiation.

[51] Benvie AM (2023). Age-dependent Pdgfrβ signaling drives adipocyte progenitor dysfunction to alter the beige adipogenic niche in male mice. Nat Commun.

[52] He C (2017). STAT1 modulates tissue wasting or overgrowth downstream from PDGFRβ. Genes Dev.

[53] Liu L (2015). Isolation of skeletal muscle stem cells by fluorescence-activated cell sorting. Nat Protoc.

[54] Richler C, Yaffe D (1970). The in vitro cultivation and differentiation capacities of myogenic cell lines. Dev Biol.

[55] Yi L, Rossi F (2011). Purification of progenitors from skeletal muscle. J Vis Exp.

[56] Sun L (1999). Design, synthesis, and evaluations of substituted 3-[(3- or 4-carboxyethylpyrrol-2-yl)methylidenyl]indolin-2-ones as inhibitors of VEGF, FGF, and PDGF receptor tyrosine kinases. J Med Chem.

[57] Li S (2005). Requirement for serum response factor for skeletal muscle growth and maturation revealed by tissue-specific gene deletion in mice. Proc Natl Acad Sci U S A.

[58] Brunetti A, Goldfine ID (1990). Role of myogenin in myoblast differentiation and its regulation by fibroblast growth factor. J Biol Chem.

[59] Schiaffino S (2015). Developmental myosins: expression patterns and functional significance. Skelet Muscle.

[60] Lewis NL (2009). Phase I study of the safety, tolerability, and pharmacokinetics of oral CP-868,596, a highly specific platelet-derived growth factor receptor tyrosine kinase inhibitor in patients with advanced cancers. J Clin Oncol.

[61] Eliazer S (2019). Wnt4 from the niche controls the mechano-properties and quiescent state of muscle stem cells. Cell Stem Cell.

[62] Olson LE, Soriano P (2011). PDGFRβ signaling regulates mural cell plasticity and inhibits fat development. Dev Cell.

[63] Saclier M (2013). Differentially activated macrophages orchestrate myogenic precursor cell fate during human skeletal muscle regeneration. Stem Cells.

[64] Webster MT, Fan C-M (2013). c-MET regulates myoblast motility and myocyte fusion during adult skeletal muscle regeneration. PLoS One.

[65] Kim S (2007). A critical function for the actin cytoskeleton in targeted exocytosis of prefusion vesicles during myoblast fusion. Dev Cell.

[66] Jahraus A (2001). ATP-dependent membrane assembly of F-actin facilitates membrane fusion. Mol Biol Cell.

[67] Kim JH (2015). Mechanisms of myoblast fusion during muscle development. Curr Opin Genet Dev.

[68] Inman GJ (2002). SB-431542 is a potent and specific inhibitor of transforming growth factor-beta superfamily type I activin receptor-like kinase (ALK) receptors ALK4, ALK5, and ALK7. Mol Pharmacol.

[69] He C (2015). PDGFRβ signalling regulates local inflammation and synergizes with hypercholesterolaemia to promote atherosclerosis. Nat Commun.

[70] Frank DA (1999). Fludarabine-induced immunosuppression is associated with inhibition of STAT1 signaling. Nat Med.

[71] Ricci F (2009). Fludarabine in the treatment of chronic lymphocytic leukemia: a review. Ther Clin Risk Manag.

[72] Gheller BJ (2019). Isolation, culture, characterization, and differentiation of human muscle progenitor cells from the skeletal muscle biopsy procedure. J Vis Exp.

[73] Minatogawa M (2017). Expansion of the phenotype of Kosaki overgrowth syndrome. Am J Med Genet A.

[74] Takenouchi T (2015). Novel overgrowth syndrome phenotype due to recurrent de novo PDGFRB mutation. J Pediatr.

[75] Benvie AM (2024). Platelet-derived growth factor receptor beta is required for embryonic specification and confinement of the adult white adipose lineage. iScience.

[76] Webb SE, Lee KK (1997). Effect of platelet-derived growth factor isoforms on the migration of mouse embryo limb myogenic cells. Int J Dev Biol.

[77] Hershey JC (2001). Revascularization in the rabbit hindlimb: dissociation between capillary sprouting and arteriogenesis. Cardiovasc Res.

[78] Verma M (2018). Muscle satellite cell cross-talk with a vascular niche maintains quiescence via VEGF and notch signaling. Cell Stem Cell.

[79] Huang P (2009). Imatinib attenuates skeletal muscle dystrophy in mdx mice. FASEB J.

[80] Contreras O (2018). Nilotinib impairs skeletal myogenesis by increasing myoblast proliferation. Skelet Muscle.

[81] Hung M (2023). The muscle stem cell niche at a glance. J Cell Sci.

[82] Duan D (2021). Duchenne muscular dystrophy. Nat Rev Dis Primers.

[83] Laumonier T, Menetrey J (2016). Muscle injuries and strategies for improving their repair. J Exp Orthop.

[84] Judson RN, Rossi FMV (2020). Towards stem cell therapies for skeletal muscle repair. NPJ Regen Med.

[85] Gheller BJ (2021). Extracellular serine and glycine are required for mouse and human skeletal muscle stem and progenitor cell function. Mol Metab.

