# Supplementary material for: PDGFRβ signaling restrains myocyte function to limit the regenerative capacity of skeletal muscle
Source: J Clin Invest. 2025 Dec 16;136(4):e188272. doi: 10.1172/JCI188272 (PMC12904705; doi:10.1172/JCI188272)
Supplement: Supplemental data [file jci-136-188272-s179.pdf]

Supplemental Figure 1

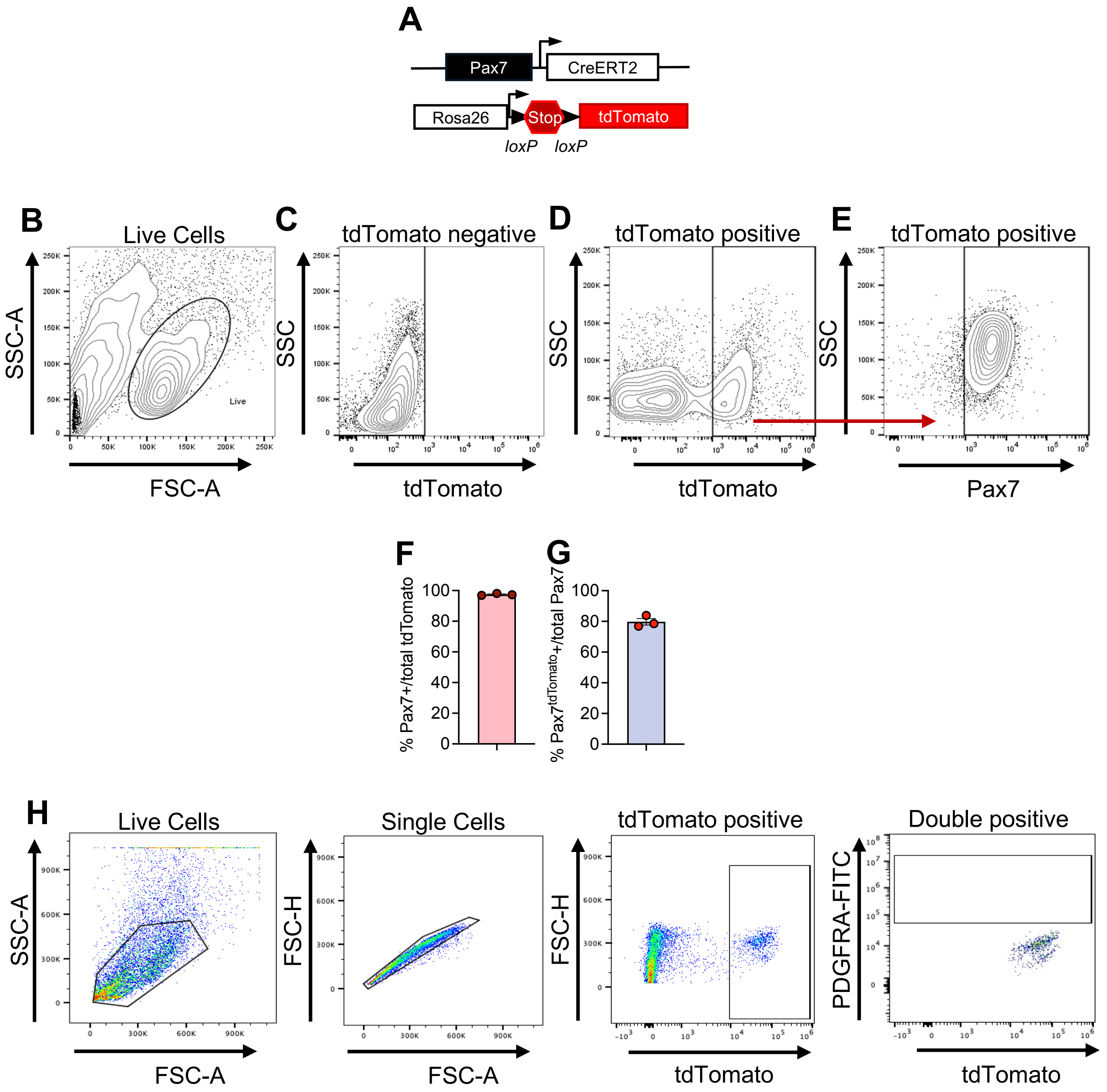

Supplementary Figure 1. Validation of the Pax7^{tdTomato} genetic tool

(A) Allelic combination to generate the TMX inducible Pax7^{tdTomato} mice.

(B) Representative FACS gating strategy for live cells.

(C) Representative FACS gating strategy for tdTomato negative cells.

(D) Representative FACS gating strategy for tdTomato positive cells.

(E) Representative FACS plots demonstrating that tdTomato positive cells are PAX7 antibody positive.

(F) FACS quantification of tdTomato cells expressing PAX7, demonstrating reporter overlapping with endogenous PAX7 expression (n = 3 biologically independent mice/group).

(G) FACS quantification of tdTomato cells out of total PAX7 antibody expressing cells, demonstrating high recombination efficiency (n = 3 biologically independent mice/group).

(H) Representative FACS plots demonstrating that tdTomato positive cells are PDGFRA negative.

Data are presented as mean values ± SEM.

Supplemental Figure 2

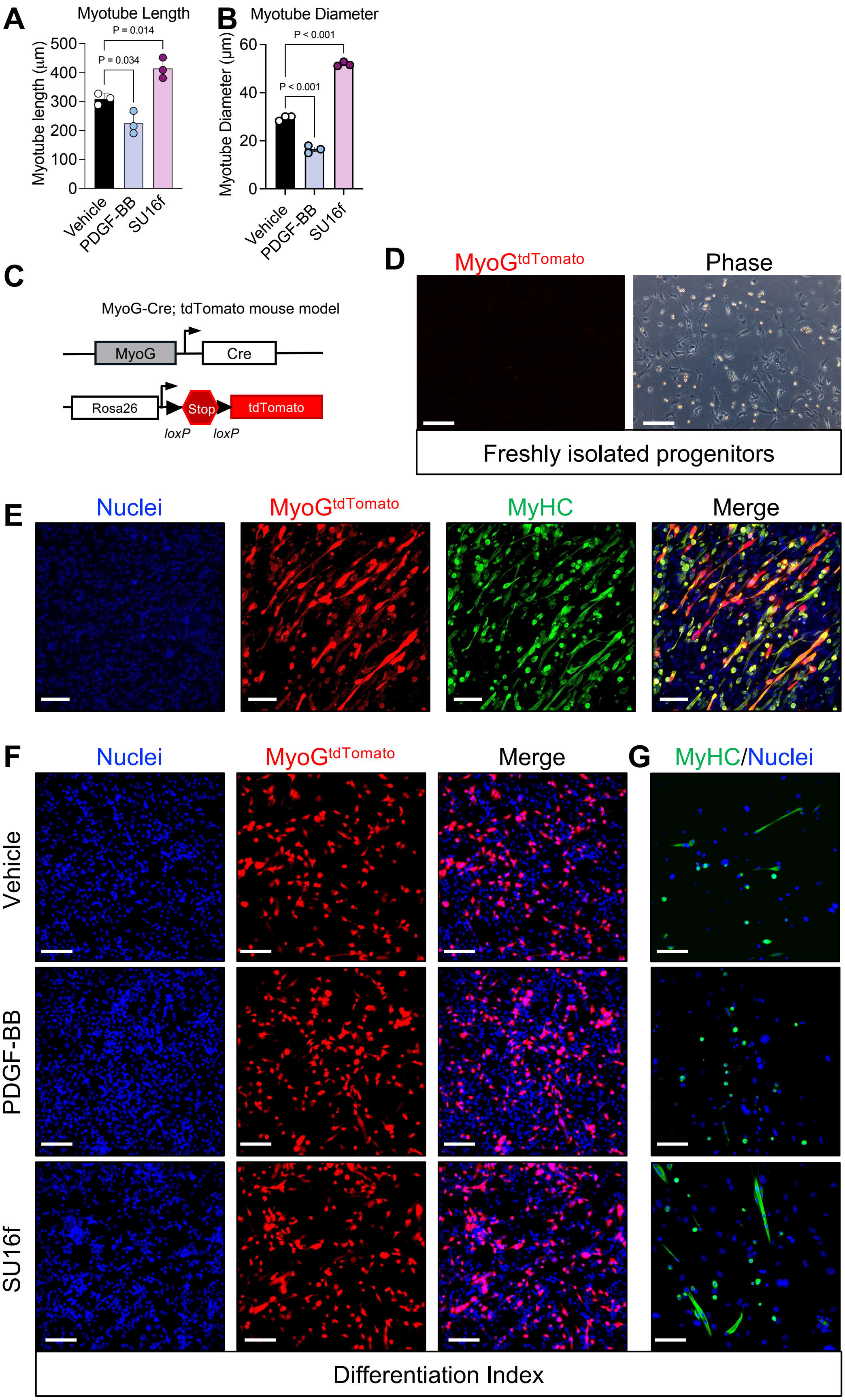

Supplementary Figure 2. PDGFRB activation alters myotube development

(A) Quantification of myotube length from cultures treated with vehicle, PDGF-BB, and SU16f (n = 3 biologically independent samples per group).

(B) Quantification of myotube diameter from cultures treated with vehicle, PDGF-BB, and SU16f (n = 3 biologically independent samples per group).

(C) Allelic combination to generate the $MyoG^{tdTomato}$ mice.

(D) Muscle progenitor cells were isolated from the hindlimb muscle groups of from $Control^{MyoG}$ and visualized for tdTomato fluorescence 72 hours after plating.

(E) Representative images of myotube cultures from $Control^{MyoG}$ mice. Cultures were visualized for tdTomato fluorescence overlap with MyHC immunostaining. Note the high correspondence between tdTomato and MyHC.

(F) Muscle progenitor cells were isolated from the hindlimb muscle groups of from $Control^{MyoG}$. Cells were plated at low density, grown, and differentiated in the presence of either vehicle (0.1% DMSO), PDGF-BB (25 ng/mL), or SU16f (1 µM). Myocyte formation was assessed by the number of $MyoG^{tdTomato}$ positive cells.

(G) Muscle progenitor cells were isolated from the hindlimb muscle groups of from $Control^{MyoG}$. Cells were plated at low density, grown, and differentiated in the presence of either vehicle (0.1% DMSO), PDGF-BB (25 ng/mL), or SU16f (1 µM). Myocyte formation was assessed by the number of MyHC positive cells.

Data represent mean ± SEM. Statistical significance was determined using a one-way ANOVA followed by Dunnett’s multiple comparison test for panels (A) and (B). Scale bar = 100 µm.

Supplemental Figure 3

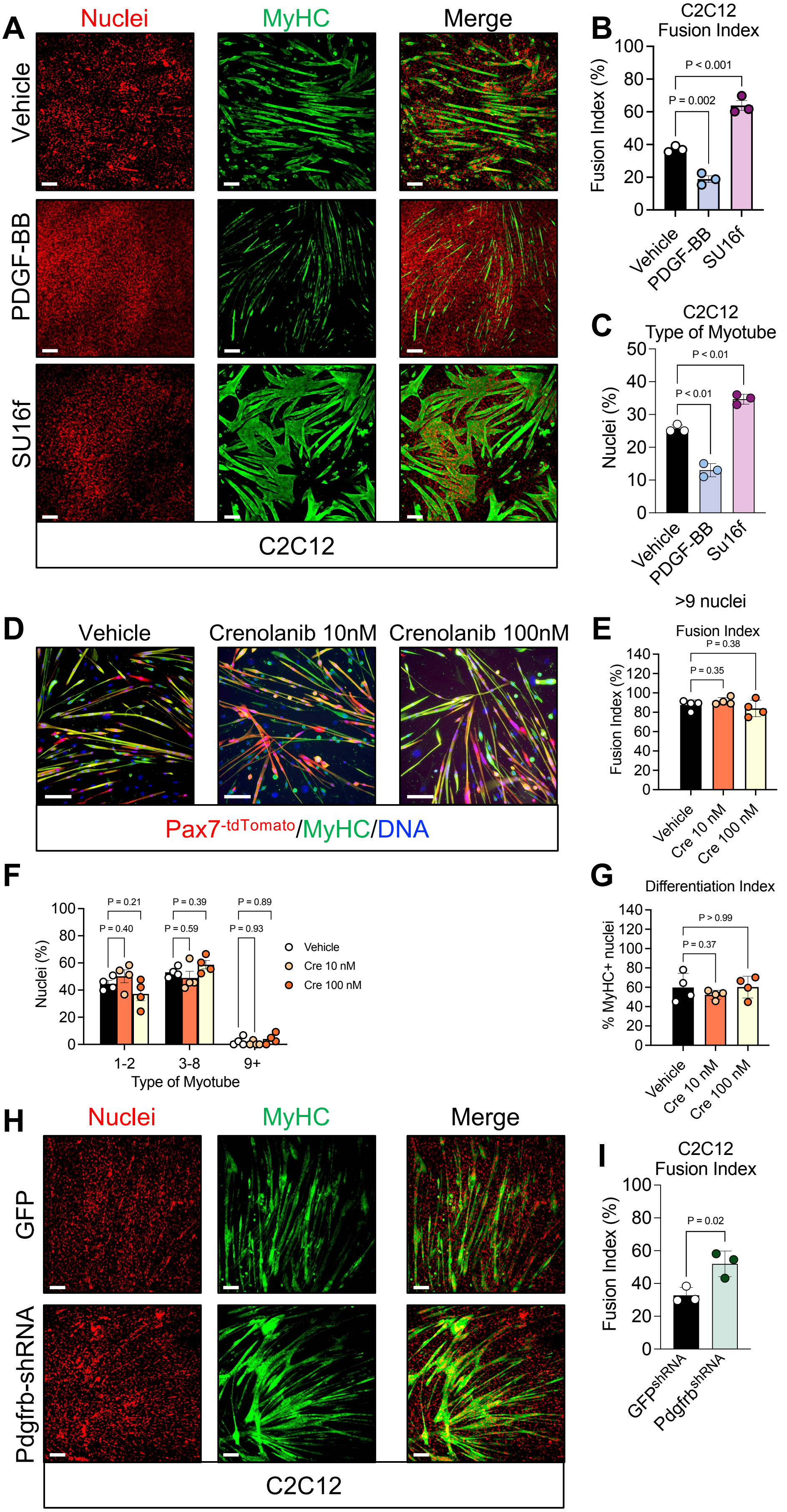

Supplementary Figure 3. PDGFRB activation alters C2C12 myotube development

(A) Representative images of C2C12 myotube development in the presence of vehicle (0.1% DMSO), PDGF-BB (25 ng/mL), or SU16f (1 μM). C2C12 derived myotubes were immunostained for MyHC to evaluate myotube development.

(B) Quantification of the fusion index from C2C12 myotube cultures described in (A), providing a measure of muscle cell fusion in response to PDGFRB activation or inhibition (n = 3 biologically independent mice per group).

(C) Quantification of myotubes with ≥9 nuclei per tube from cultures described in (A) (n = 3 biologically independent mice per group).

(D) Hindlimb muscles were collected from P30 $Pax7^{tdTomato}$ male mice. Isolated muscle progenitor cells were given TMX and cultured. Throughout differentiation, cells were treated with vehicle or crenolanib, a selective PDGFRA inhibitor (10 or 100 nM), for five days and then myotube formation was assessed. Representative images of MyHC showing myotube development, providing visual evidence of crenolanib on myotube formation.

(E) Quantification of the fusion index of differentiated cultures described in (D) (n = 4 biologically independent mice per group).

(F) Quantification of myotube nuclei count and distribution from cultures described in (D) (n = 4 biologically independent mice per group).

(G) Treated cells (vehicle or crenolanib (10 or 100 nM)) described in (D), were scored for MyHC positivity to determine differentiation index (n = 4 biologically independent mice per group).

(H) Representative images of C2C12 myotube development in the presence of GFP- or *Pdgfrb*-shRNA. C2C12 derived myotubes were immunostained for MyHC to evaluate myotube development.

(I) Quantification of the fusion index from C2C12 myotube cultures knocked downed for *Pdgfrb* described in (H), providing a measure of muscle cell fusion in response to *Pdgfrb* knockdown (n = 3 biologically independent mice per group).

Data represent mean ± SEM. Statistical significance was determined using a one-way ANOVA ((B), (C)) or two-way ANOVA ((E), (F), (G)) followed by Dunnett's multiple comparison test. Unpaired Student's *t* test for panel (I). Scale bar = 100 μm.

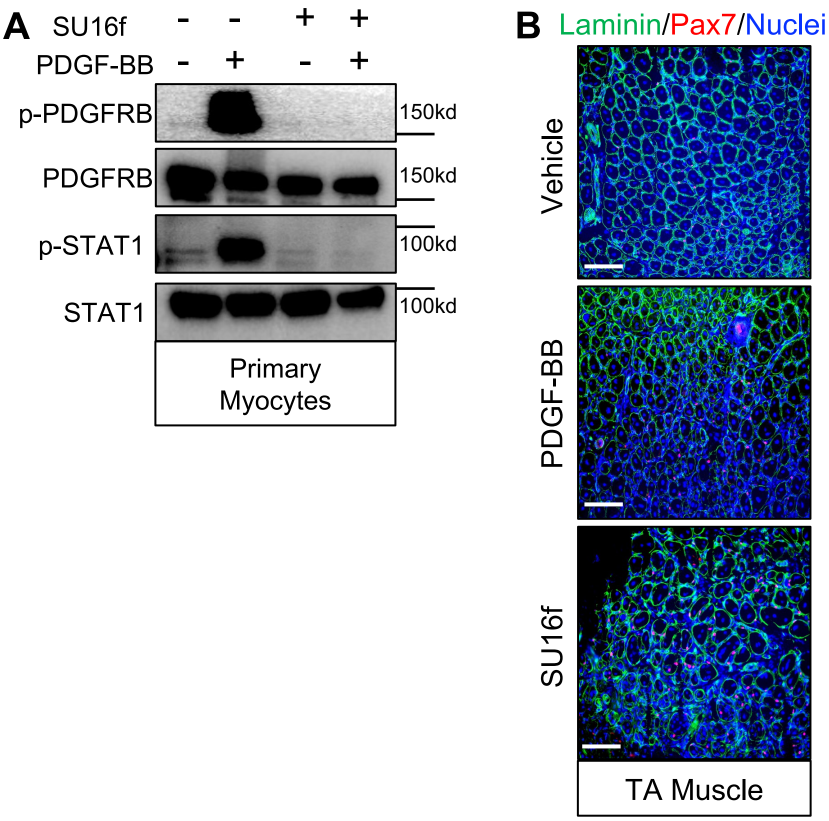

Supplementary Figure 4. PDGFRB activation in muscle

(A) Representative immunoblot analysis of phosphorylated and total PDGFRB and STAT1 levels in myocytes treated with vehicle or PDGF-BB (15 ng/mL for 15 min) and co-treated with SU16f (1 µM for 2 hours).

(B) $MyoG^{tdTomato}$ mice were subjected to a single intramuscular injection of 1.2% $BaCl_2$ to induce injury in the TA muscle. Subsequently, mice were administered one dose of vehicle, PDGF-BB (50 ng/mouse), or SU16f (2 mg/Kg) for five consecutive days by intraperitoneal injection. Representative images of laminin and PAX7 staining of TA muscle sections at seven d.p.i.

Scale bar = 100 µm.

Supplemental Figure 5

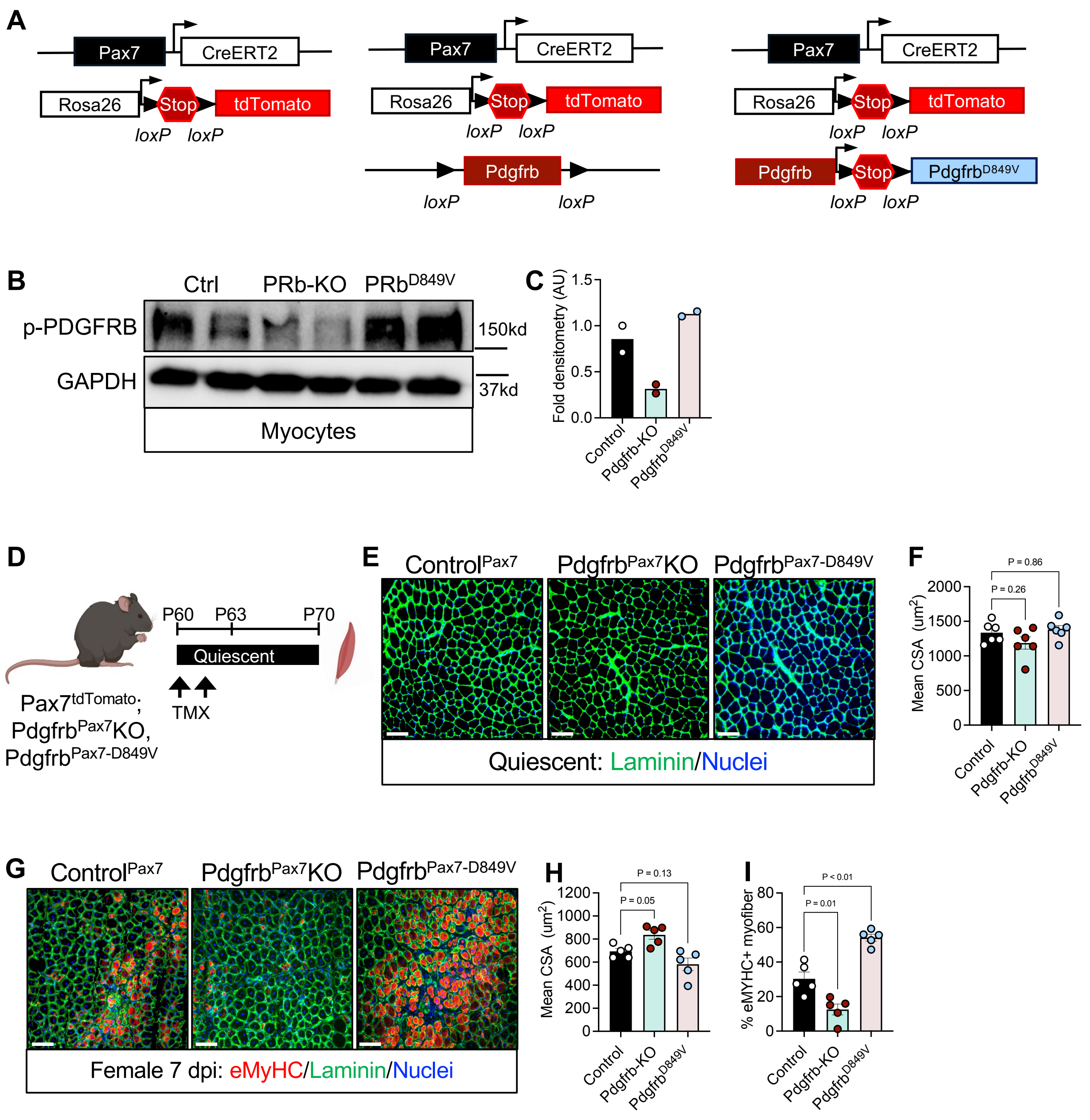

Supplementary Figure 5. Genetically altering *Pdgfrb* expression changes muscle regeneration

(A) Schematic of allelic combination to generate $Control^{Pax7}$, $Pdgfrb^{Pax7}KO$, and $Pdgfrb^{Pax7\text{-}D849V}$ mice

(B) Representative immunoblot analysis of phosphorylated PDGFRB in cultured myocytes from $Control^{Pax7}$, $Pdgfrb^{Pax7}KO$, and $Pdgfrb^{Pax7\text{-}D849V}$ mice (n =2 biologically independent mice per group).

(C) Quantification of the immunoblot described in (B) (n =2 biologically independent mice per group).

(D) Schematic of the experimental approach. $Control^{Pax7}$, $Pdgfrb^{Pax7}KO$, and $Pdgfrb^{Pax7\text{-}D849V}$ mice were administered TMX at postnatal day 60 (P60) to induce Cre-mediated recombination. TA muscles were harvested at P70.

(E) Representative images of laminin immunostaining of quiescent TA muscle sections from mice described in (D).

(F) Quantification of mean cross-sectional area (CSA) of TA myofibers from sections described in (E) from mice described in (D) (n = 6 biologically independent mice per group).

(G) $Control^{Pax7}$, $Pdgfrb^{Pax7}KO$, and $Pdgfrb^{Pax7\text{-}D849V}$ female mice were administered TMX at postnatal day 60 (P60) to induce Cre-mediated recombination. TA muscles were intramuscular injected with 1.2% $BaCl_2$ to induce injury and analyzed at seven days later. Representative images of injured TA muscle sections immunostained for eMyHC and laminin from mice described in (A), illustrating the regenerative response is not sex-dependent.

(H) Quantification of mean CSA of TA myofibers from female mice and sections described in (G) (n = 5 biologically independent mice per group).

(I) Quantification of eMyHC immunostaining from TA muscle sections described in (G), indicating the level of ongoing regeneration post-injury (n = 5 biologically independent mice per group).

Data are presented as mean values with individual data points ± S.E.M. Statistical significance was determined using a one-way ANOVA followed by Dunnett's multiple comparison test for panels (F), (H), and (I). Scale bar = 100 μm.

Supplemental Figure 6

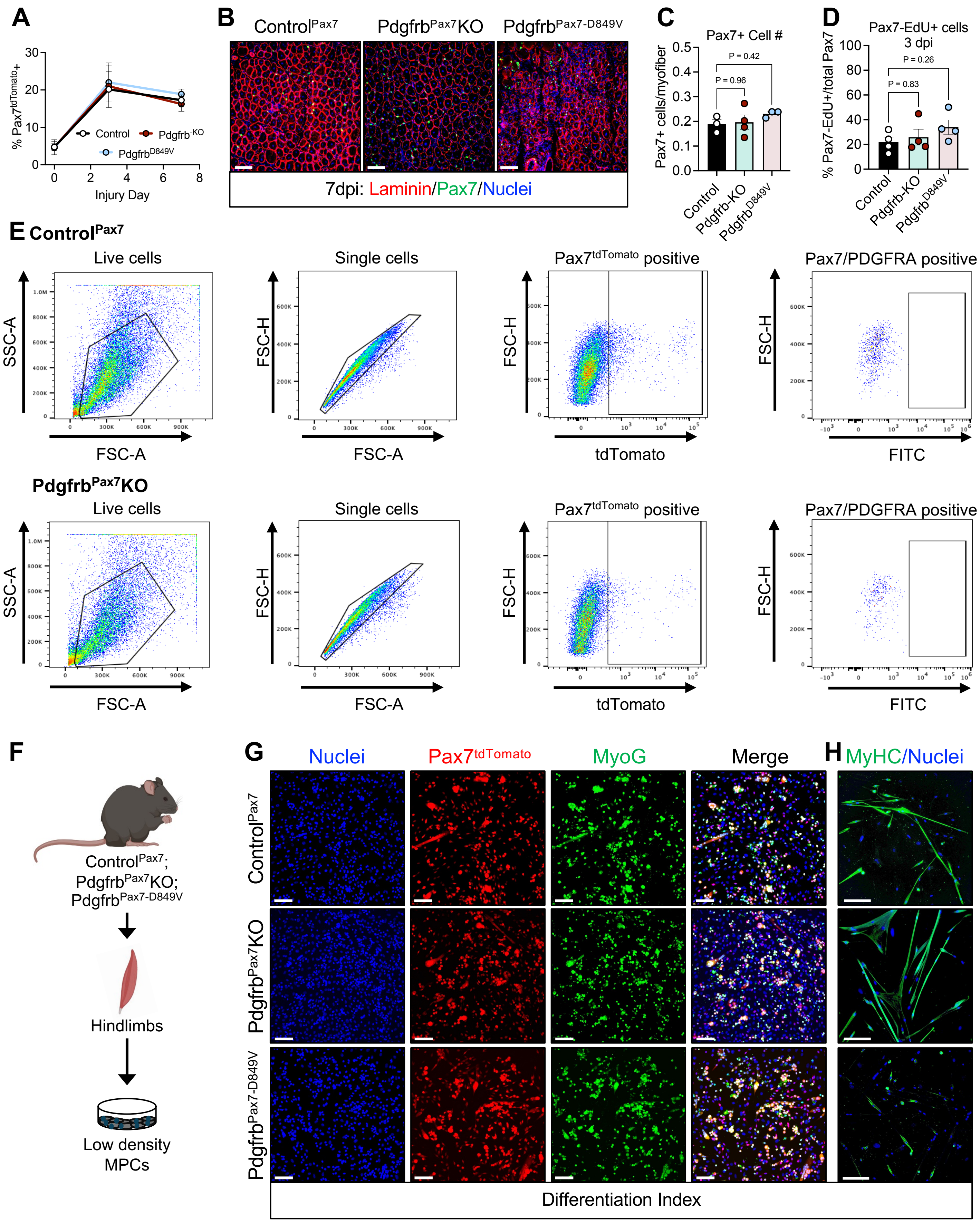

Supplementary Figure 6. Genetically altering *Pdgfrb* expression does not change muscle progenitor cellular kinetics

(A) Control^{Pax7}, Pdgfrb^{Pax7}KO, and Pdgfrb$^{Pax7\text{-}D849V}$ male mice were administered TMX at postnatal day 60 (P60) to induce Cre-mediated recombination. TA muscles were injured with 1.2% $BaCl_2$ and Pax7^{tdTomato}+ cells were FACS at zero, three, and seven d.p.i. and total Pax7^{tdTomato} cell number was evaluated (n = 4-5 biologically independent mice/group).

(B) Representative images of injured TA muscle sections at seven d.p.i. immunostained for PAX7 from mice described in (A).

(C) Quantification of PAX7 immunostaining from TA muscle sections described in (B), indicating the number of PAX7+ cells within ongoing regeneration muscle across different genotypes (n = 3-4 biologically independent mice per group).

(D) Control^{Pax7}, Pdgfrb^{Pax7}KO, and Pdgfrb$^{Pax7\text{-}D849V}$ male mice were administered TMX at postnatal day 60 (P60) to induce Cre-mediated recombination. TA muscles were injured with 1.2% $BaCl_2$. At three dpi, mice were administered EdU for eight hours. Pax7^{tdTomato}+ cells were FACS isolated from TA muscle groups and evaluated for EdU incorporation and quantified (n = 4 biologically independent mice per group).

(E) Representative flow cytometric plots of PDGFRA staining within Pax7^{tdTomato} cells from TA muscle groups from mice Control^{Pax7} and Pdgfrb^{Pax7}KO described in (D) (n = 4 biologically independent mice per group).

(F) Schematic of the experimental approach. Muscle progenitors were isolated from hindlimb muscle groups from of Control^{Pax7}, Pdgfrb^{Pax7}KO, and Pdgfrb$^{Pax7\text{-}D849V}$. After isolation, low density cultures were administered TMX to induce recombination, expanded, and subsequently differentiated and differentiation index was assessed.

(G) Representative images of colocalization of tdTomato fluorescence and myogenin-positive cells, used to calculate the differentiation index from mice and cells described in (F).

(H) Representative images of MyHC staining used to determine the differentiation status from mice and cells described in (F).

Data are presented as mean values ± S.E.M. Statistical significance was determined using a one-way ANOVA followed by Dunnett's multiple comparison test for panels (C) and (D). Scale bar = 100 μm.

Supplemental Figure 7

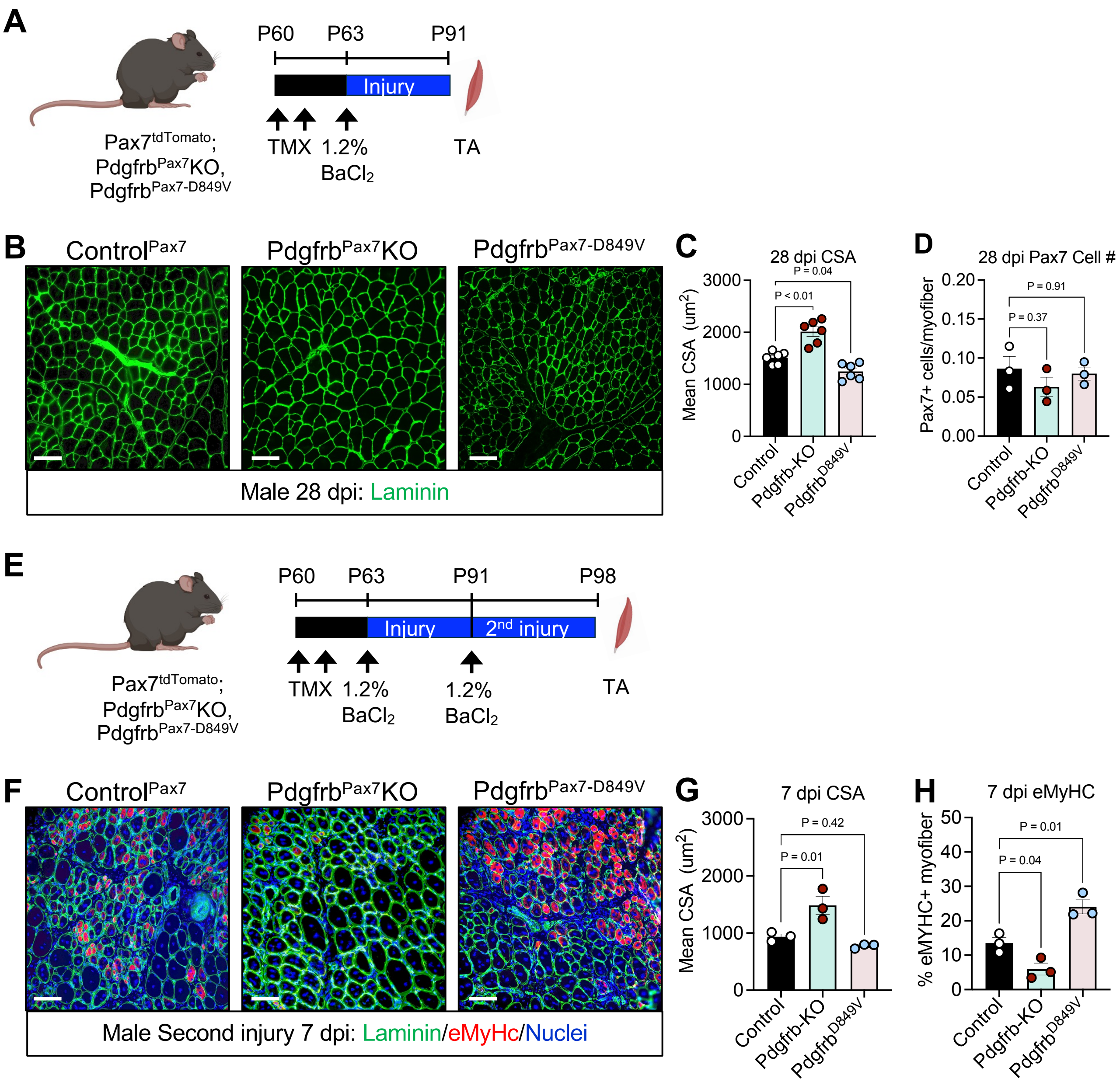

Supplementary Figure 7. Genetically altering *Pdgfrb* expression changes muscle regeneration.

(A) Schematic of the experimental approach. Control^{Pax7}, Pdgfrb^{Pax7}KO, and Pdgfrb$^{Pax7\text{-}D849V}$ mice were administered TMX at postnatal day 60 (P60) to induce Cre-mediated recombination. TA muscles were subsequently injured with 1.2% $BaCl_2$ and analyzed at 28 d.p.i.

(B) Representative images of laminin immunostaining of TA muscle sections at 28 d.p.i. from injured mice described in (A).

(C) Quantification of mean CSA of injured TA myofibers at 28 dpi from sections described in (B) from mice described in (A) (n = 6 biologically independent mice per group).

(D) Quantification of PAX7 immunostaining from TA muscles from mice described in (A) at 28 dpi (n = 3 biologically independent mice per group).

(E) Schematic of the experimental approach. Control^{Pax7}, Pdgfrb^{Pax7}KO, and Pdgfrb$^{Pax7\text{-}D849V}$ mice were administered TMX at postnatal day 60 (P60) to induce Cre-mediated recombination. TA muscles were subsequently injured with 1.2% $BaCl_2$ and allowed to recover for 28 days. TA muscle were re-injured with 1.2% Ba Cl_2 and analyzed at seven d.p.i for muscle regeneration.

(F) Representative images of laminin and eMyHC immunostaining of re-injured TA muscle sections at seven d.p.i. from mice described in (E).

(G) Quantification of mean CSA of re-injured TA myofibers at seven dpi from sections described in (F) from mice described in (E) (n = 3 biologically independent mice per group).

(H) Quantification of eMyHC immunostaining from re-injured TA muscle sections described in (F), indicating the level of ongoing regeneration post-re-injury (n = 3 biologically independent mice per group).

Data are presented as mean values ± S.E.M. Statistical significance was determined using a one-way ANOVA followed by Dunnett's multiple comparison test for panels (C), (D), (G) and (H). Scale bar = 100 μm.

Supplemental Figure 8

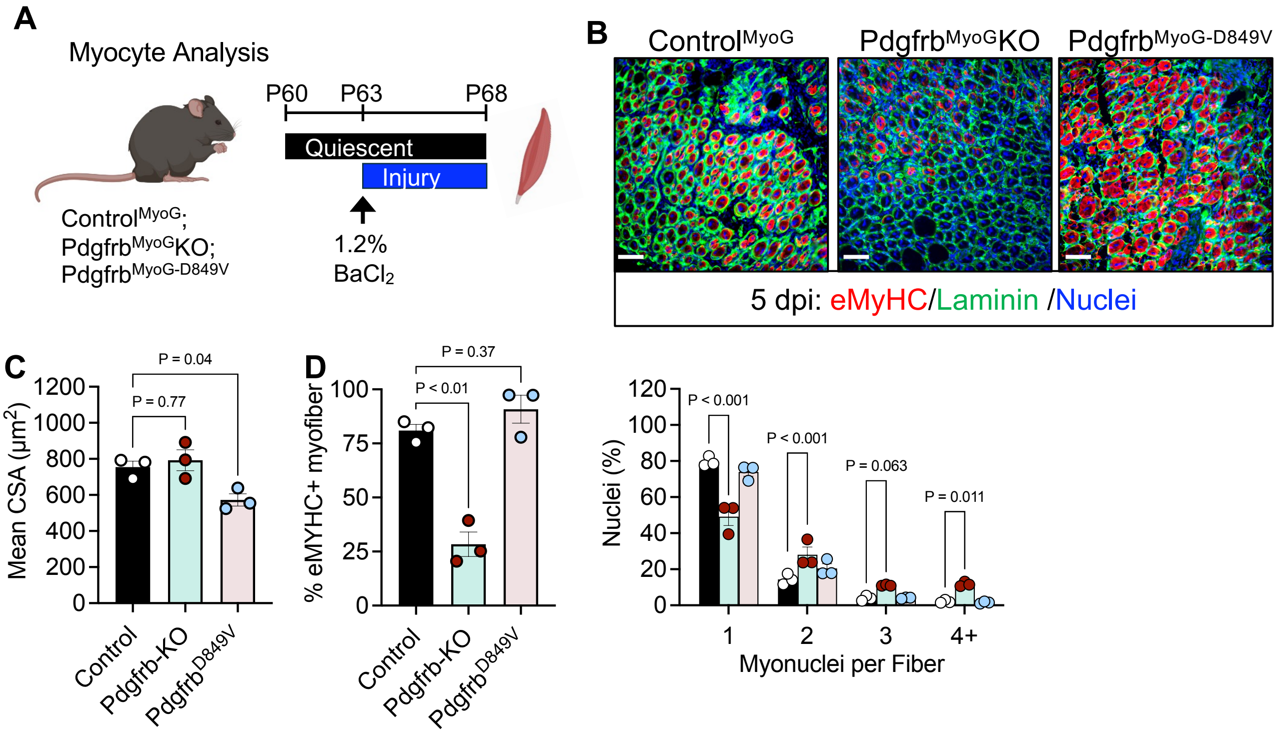

Supplementary Figure 8. PDGFRB regulates myocyte function to control skeletal muscle regeneration

(A) Experimental design: TA muscles from Control^{MyoG}, Pdgfrb^{MyoG}KO, and Pdgfrb$^{MyoG\text{-}D849V}$ mice were subjected to chemical injury using 1.2% $BaCl_2$. Muscle tissues were harvested and analyzed at five days later.

(B) Representative images of injured TA muscle sections immunostained for eMyHC and laminin from mice described in (A), illustrating the regenerative response across different genotypes at five days post injury.

(C) Quantification of mean cross-sectional area (CSA) of injured TA myofibers from sections described in (B) from mice described in (A) (n = 3 biologically independent mice per group).

(D) Quantification of eMyHC-positive fibers within the injured TA muscle from sections described in (B) from mice described in (A) (n = 3 biologically independent mice per group).

(E) Quantification of myonuclear accretion within injured myofibers from TA muscle sections described in (B) from mice described in (A), providing insights into cellular fusion and muscle repair dynamics in the different experimental groups (n = 3 biologically independent mice per group).

Data are presented as mean values ± S.E.M. Statistical significance was determined using a one-way ANOVA for panels (C) and (D) or two-way ANOVA for panel (E) followed by Dunnett's multiple comparison test. Scale bar = 100 μm.

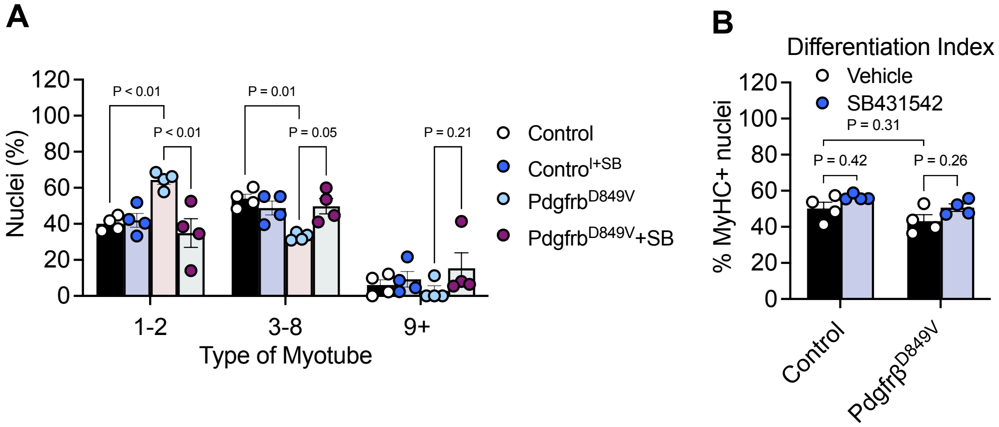

Supplementary Figure 9. PDGFRB cooperates with the TGFB signaling pathway

(A) Quantification of myotube nuclei count and distribution in the presence of TGFB signaling inhibition. Muscle progenitors isolated from hindlimb muscle groups from $Control^{Pax7}$ and $Pdgfrb^{Pax7\text{-}D849V}$. Cells were given TMX, grown, and differentiated in the presence of vehicle or SB431542 (5 µM) (n = 4 biologically independent mice per group).

(B) $Control^{Pax7}$ and $Pdgfrb^{Pax7\text{-}D849V}$ muscle progenitors described in (A) were scored for MyHC positivity to determine differentiation index (n = 4 biologically independent mice per group).

Data are presented as mean ± S.E.M. Statistical significance was determined using a two-way ANOVA followed by Tukey's multiple comparison test for panels (A) and (B).

Supplemental Figure 10

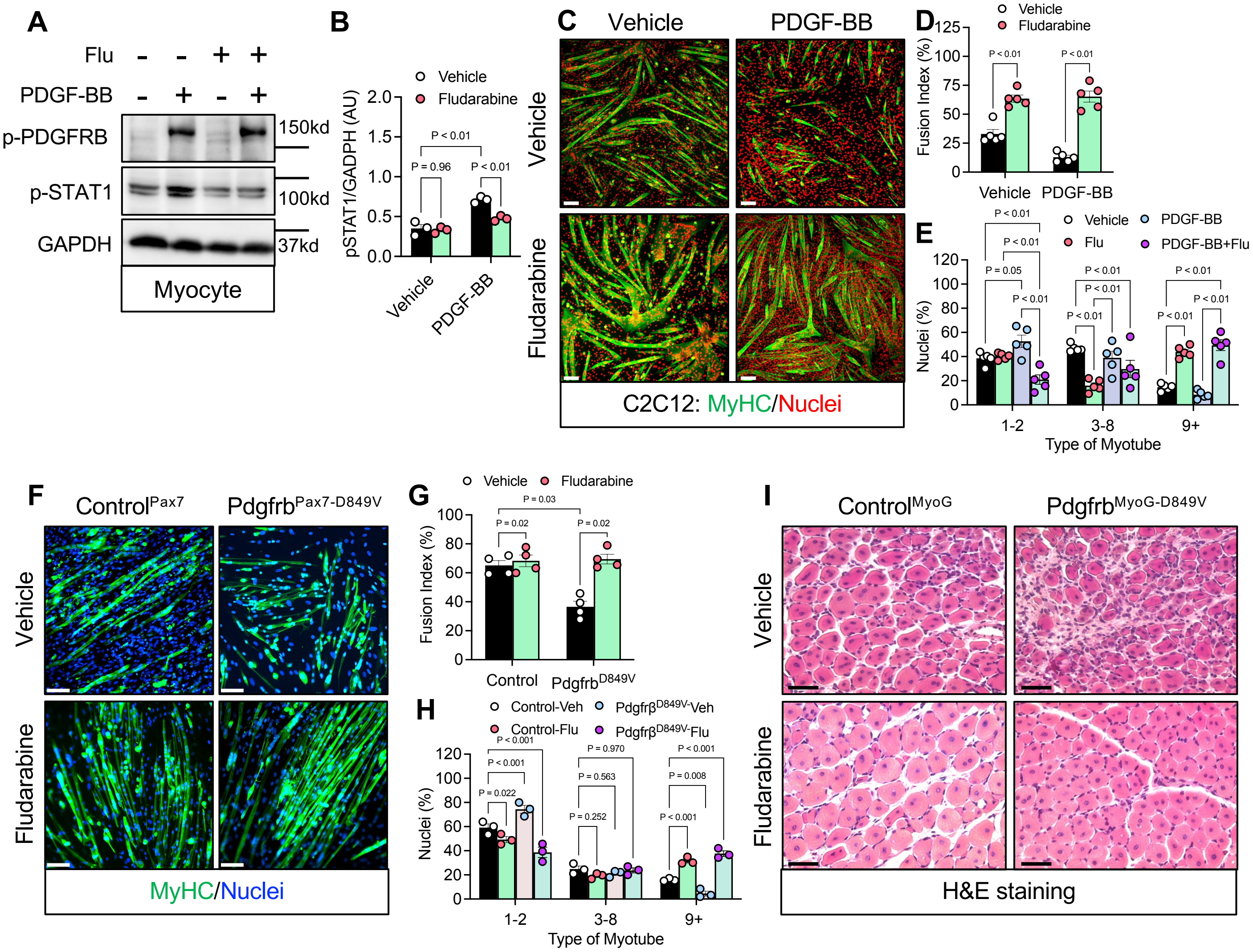

Supplementary Figure 10. STAT1 mediates PDGFRβ signaling to mediate myocyte fusion

(A) Muscle progenitor cells were isolated from the hindlimb muscle groups of $Control^{Pax7}$ male mice. After isolation, cells were expanded and subsequently differentiated for one day. Myocytes were treated with vehicle, PDGF-BB (15 ng/mL), fludarabine (1 μM), or the combination of PDGF-BB and fludarabine for 15 minutes. Representative immunoblot of phosphorylated PDGFRB and STAT1.

(B) Quantification of independent immunoblots described in (A) (n = 3 biologically independent mice/group).

(C) Representative of images of C2C12 myotube development in the presence of vehicle (0.1% DMSO), PDGF-BB (25 ng/mL), fludarabine (1 μM) or the combination of PDGF-BB and fludarabine. C2C12 derived myotubes were immunostained for MyHC to evaluate myotube development.

(D) Quantification of the fusion index from C2C12 myotube cultures described in (C), providing a measure of muscle cell fusion in response to STAT1 inhibition (n = 5 biologically independent mice per group).

(E) Quantification of myotube nuclei and distribution per tube type from C2C12 cultures described in (C) (n = 5 biologically independent mice per group).

(F) Muscle progenitor cells were isolated from the hindlimb muscle groups of $Control^{Pax7}$ and $Pdgfrb^{Pax7\text{-}D849V}$ male mice. After isolation, cells were administered TMX to induce recombination, expanded, and differentiated in the presence of vehicle (0.1% DMSO) or fludarabine (1 μM) for five days. Subsequently, myotube development was assessed.

(G) Quantification of the fusion index from the images described in (F), reflecting the efficiency of muscle cell fusion into myotubes (n = 4 biologically independent mice per group).

(H) Quantification of myotube nuclei count and distribution from the images described in (F) (n = 3 biologically independent mice per group).

(I) Representative images of hematoxylin and eosin (H&E) staining of injured TA muscle sections from $Control^{MyoG}$ and $Pdgfrb^{MyoG\text{-}D849V}$ treated with vehicle or fludarabine for five days.

Data are presented as mean values ± S.E.M. Statistical significance was determined using a two-way ANOVA with Tukey's (B), (D), (E), or Dunnett's (H), or Šídák's (G) multiple comparison tests. Scale bar = 100 μm.

Supplemental Figure 11

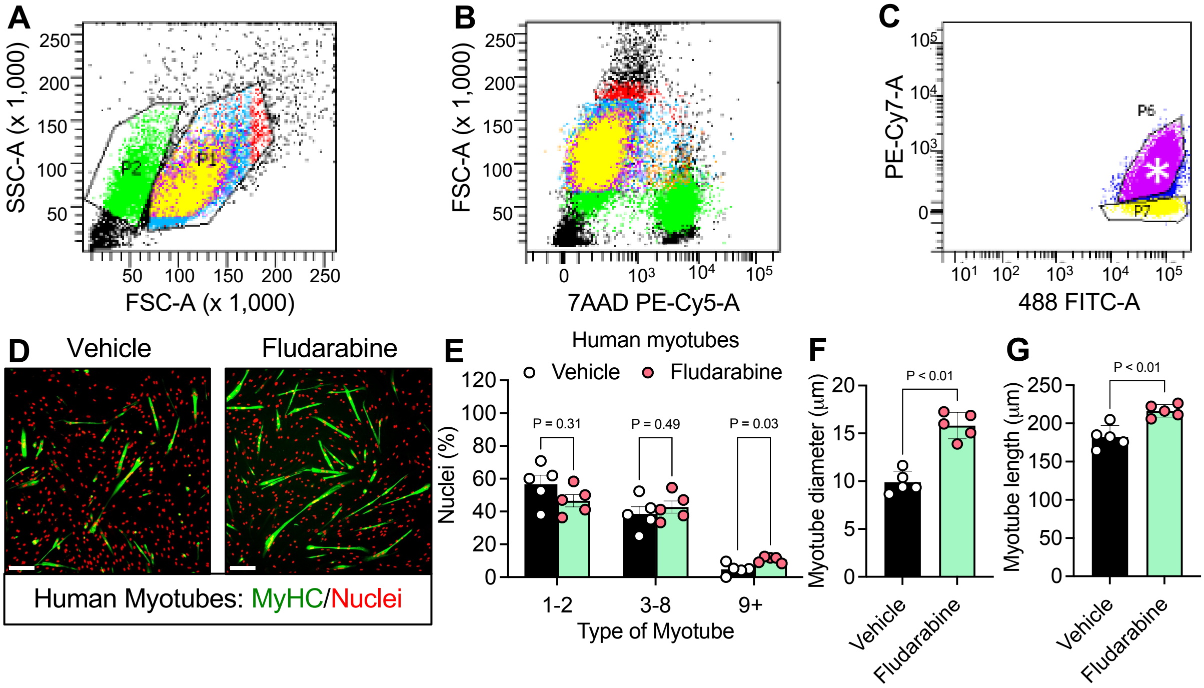

Supplementary Figure 11. Modulating PDGFRB signaling regulates human myocyte fusion

(A) Representative flow cytometric plots of side and forward scatter area on live cells from human muscle samples.

(B) Representative flow cytometric plot of viability staining from isolated human muscle cells.

(C) Representative flow cytometric plots on positive selection markers CD56 and CD29.

(D) Representative images of human myotube development in cultures treated with vehicle (0.1% DMSO) or fludarabine (1 µM).

(E) Quantification of myotube nuclei and distribution from cultures described in (D) (n = 5 biologically independent humans/group).

(F) Quantification of myotube diameter from cultures described in (D) (n = 5 biologically independent humans/group).

(G) Quantification of myotube length from cultures described in (D) (n = 5 biologically independent humans/group).

Data are presented as mean values with individual data points ± S.E.M. Statistical significance was determined using a multiple unpaired Students *t* test with Holm-Sidak correction (E) and unpaired Students *t* test for panels (F) and (G). Scale bar = 100 µm.
