# Supplementary figures and images for: PDGFRβ signaling restrains myocyte function to limit the regenerative capacity of skeletal muscle
Source: J Clin Invest. 2025 Dec 16;136(4):e188272. doi: 10.1172/JCI188272 (PMC12904705; doi:10.1172/JCI188272)

Main Figure 1F.

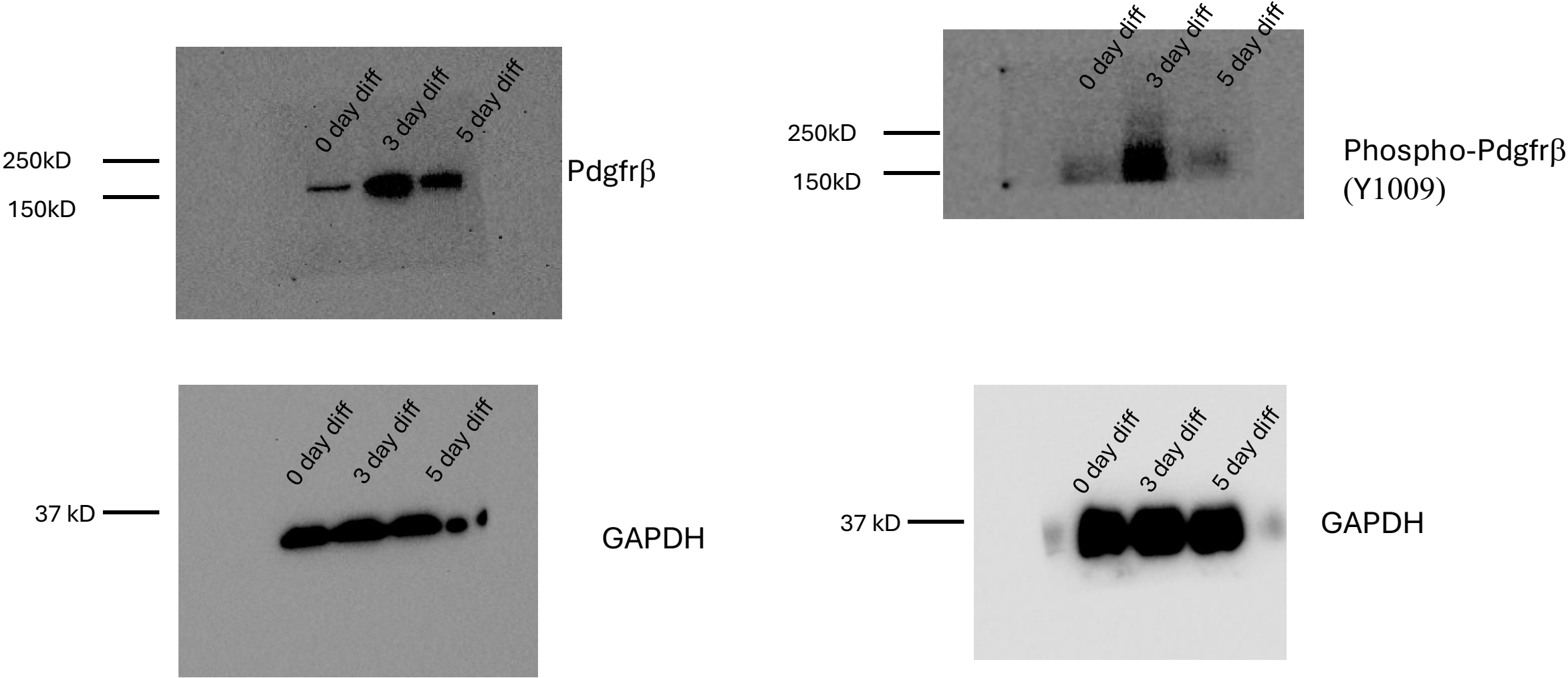

# Main Figure 1G

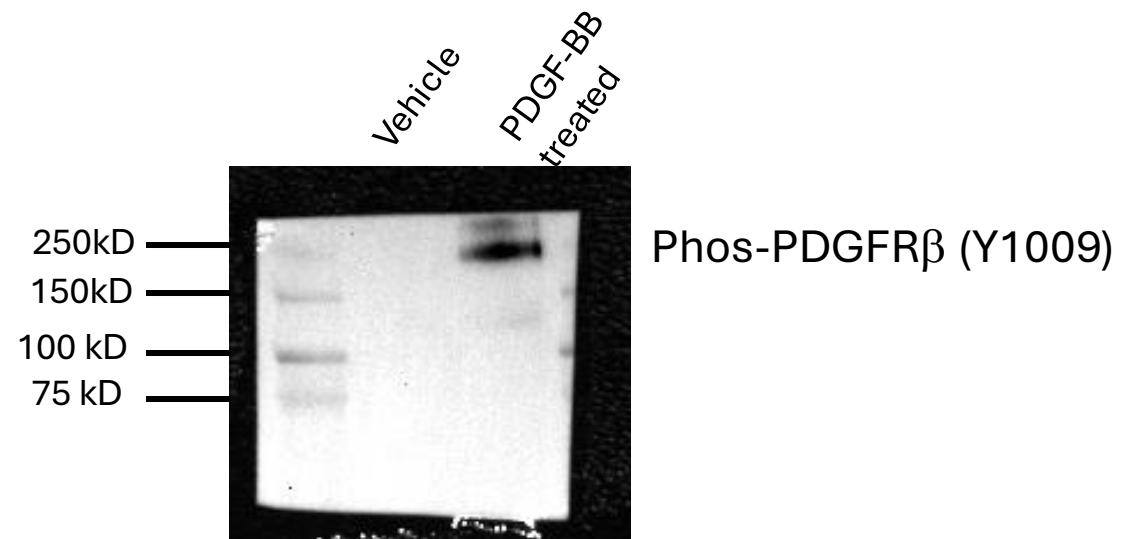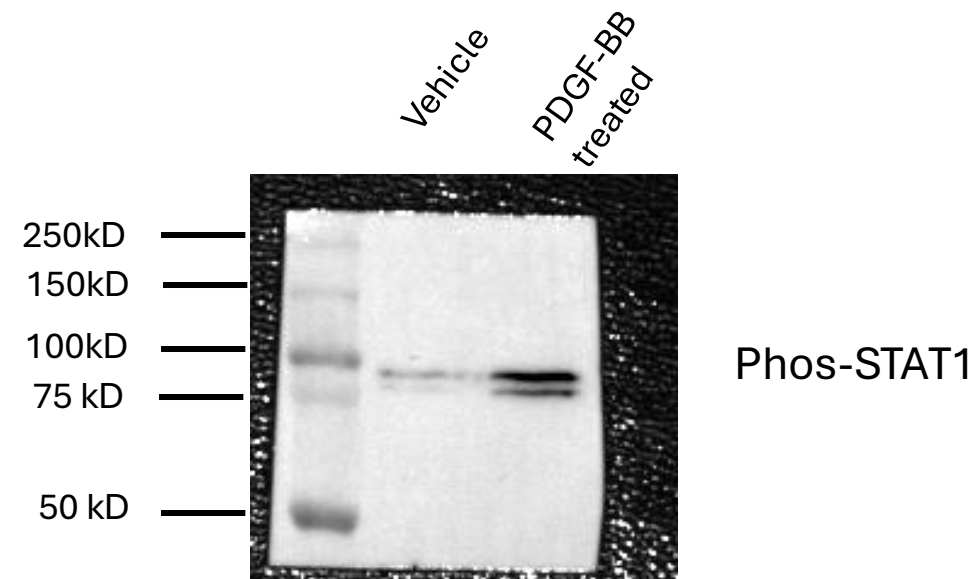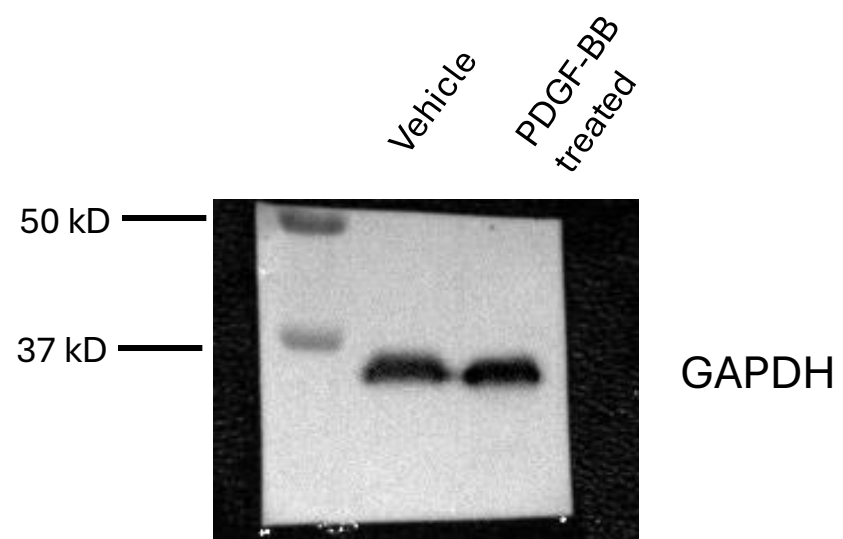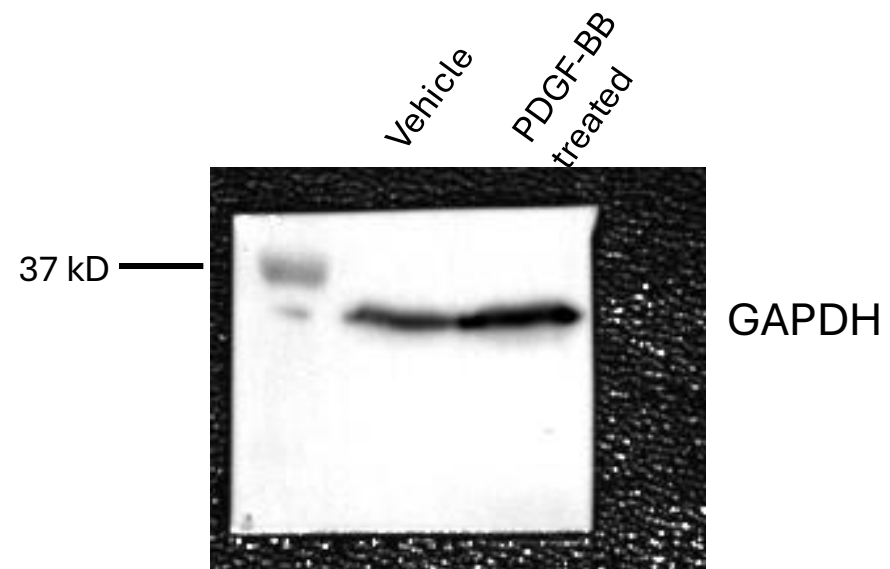

Supplemental Figure 4A

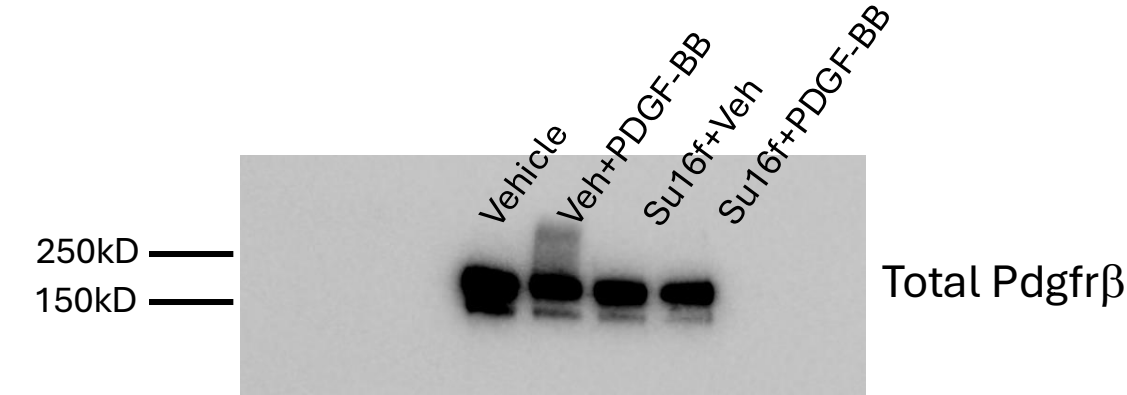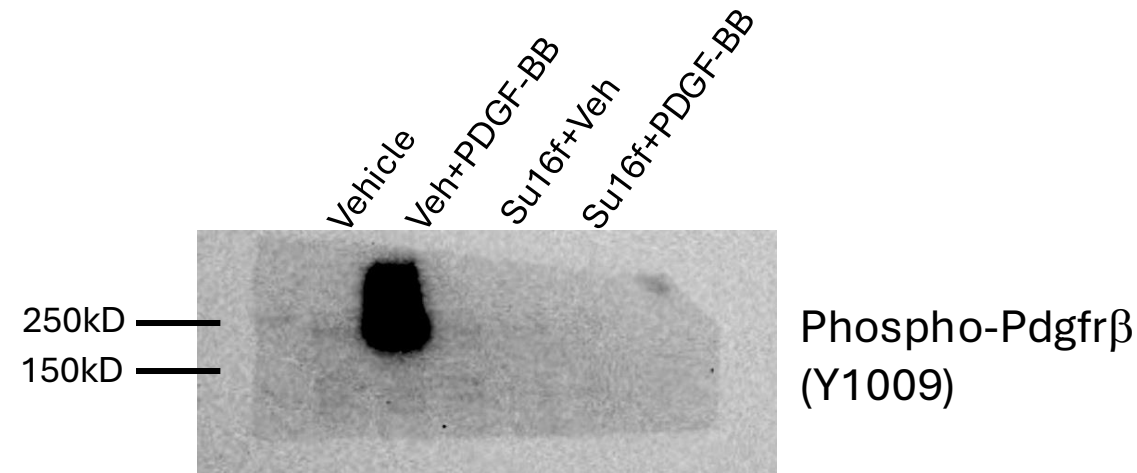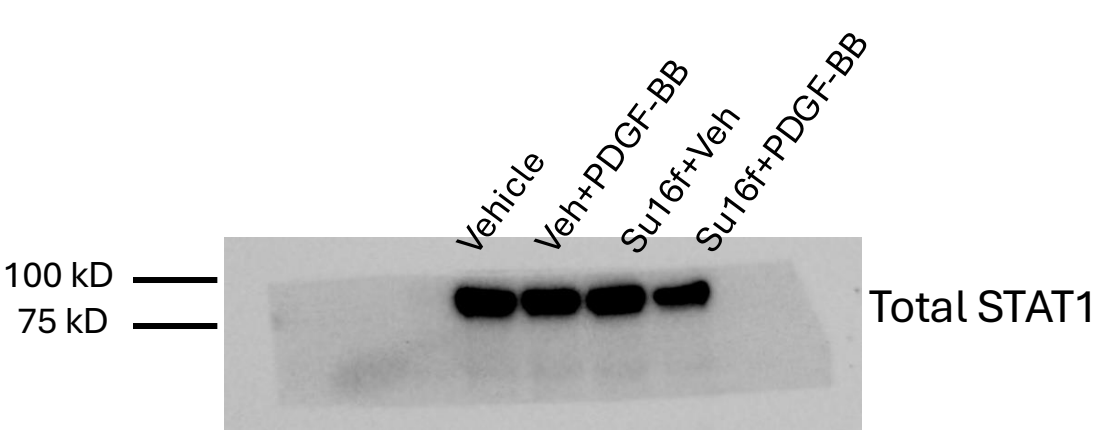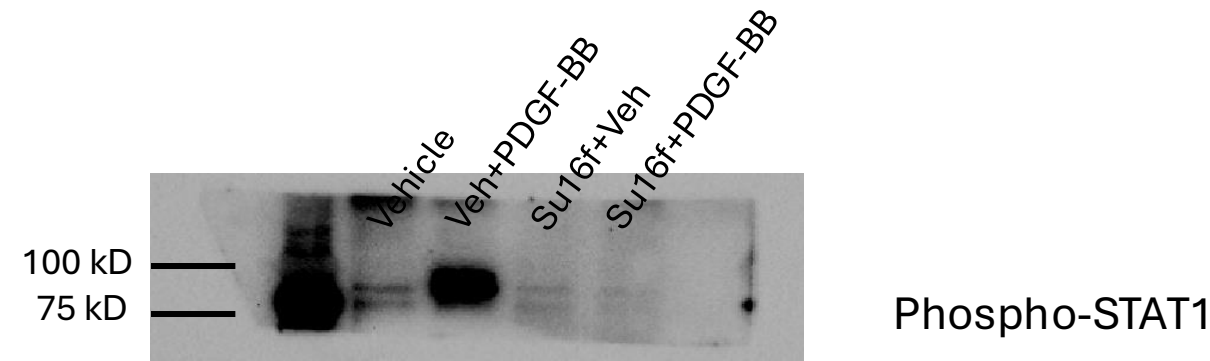

# Supplemental 5B.

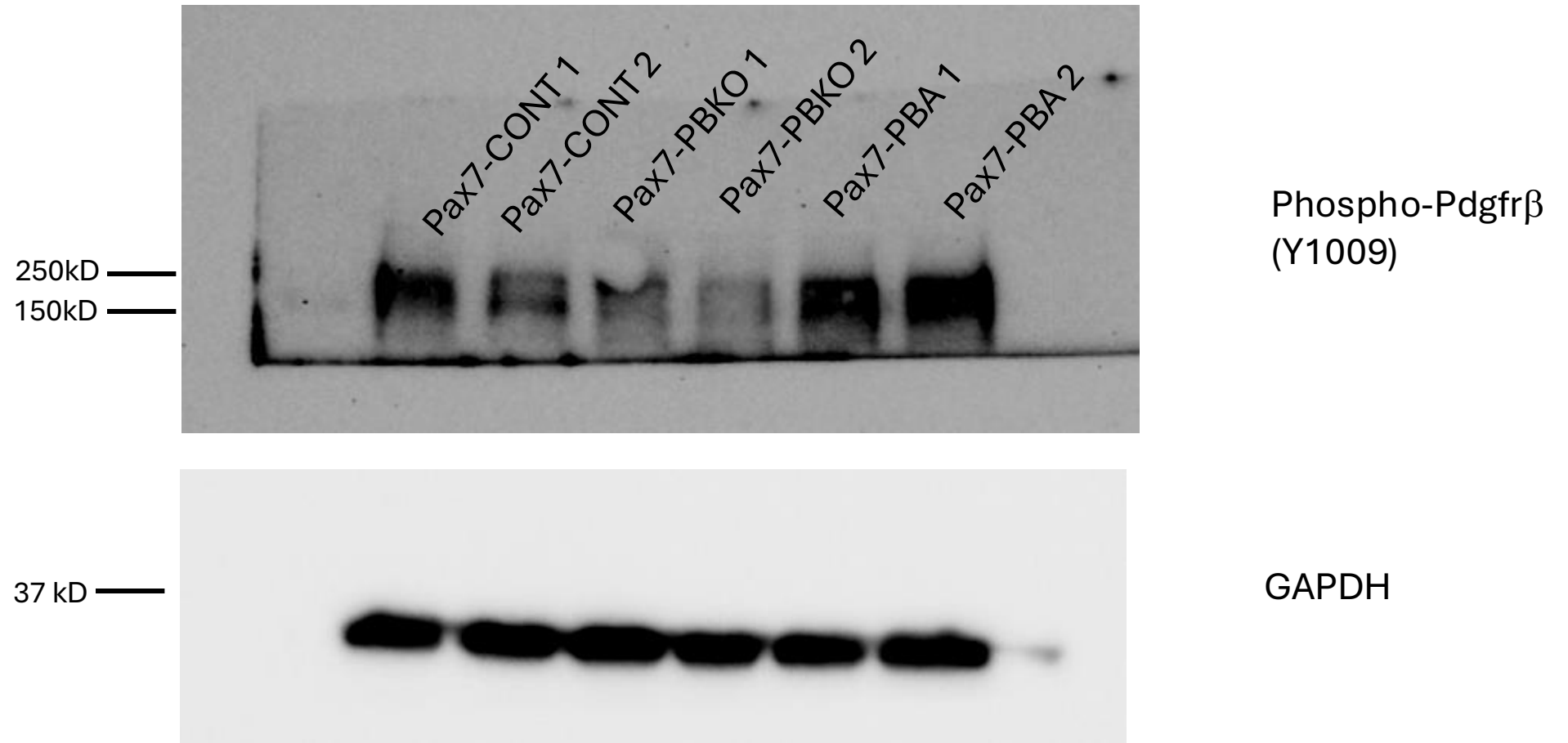

Supplemental Figure 10A

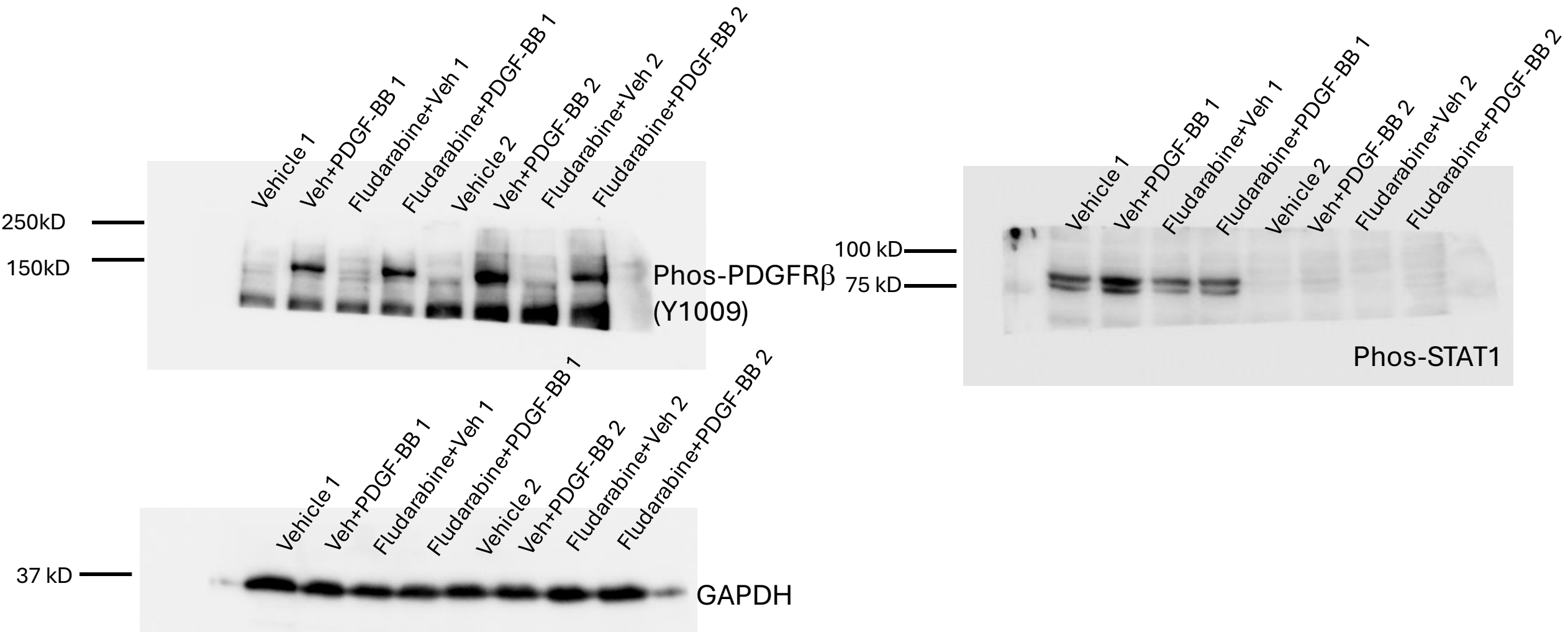

Supplement: Unedited blot and gel images [file jci-136-188272-s180.pdf]
